# Comparative Genomics of Non-TNL Disease Resistance Genes from Six Plant Species

**DOI:** 10.3390/genes8100249

**Published:** 2017-09-30

**Authors:** Madhav P. Nepal, Ethan J. Andersen, Surendra Neupane, Benjamin V. Benson

**Affiliations:** 1 Department of Biology and Microbiology, South Dakota State University, Brookings, SD 57007, USA; Ethan.Andersen@sdstate.edu (E.J.A.); Surendra.Neupane@sdstate.edu (S.N.); 2 Sanford PROMISE, Sanford Research, Sioux Falls, SD 57104, USA; Benjamin.Benson@Sanfordhealth.org

**Keywords:** nucleotide-binding site, leucine-rich repeat, NBS-LRR, evolutionary divergence, gene clustering, gene duplication, R genes, plant defense

## Abstract

Disease resistance genes (R genes), as part of the plant defense system, have coevolved with corresponding pathogen molecules. The main objectives of this project were to identify non-Toll interleukin receptor, nucleotide-binding site, leucine-rich repeat (nTNL) genes and elucidate their evolutionary divergence across six plant genomes. Using reference sequences from *Arabidopsis*, we investigated nTNL orthologs in the genomes of common bean, *Medicago*, soybean, poplar, and rice. We used Hidden Markov Models for sequence identification, performed model-based phylogenetic analyses, visualized chromosomal positioning, inferred gene clustering, and assessed gene expression profiles. We analyzed 908 nTNL R genes in the genomes of the six plant species, and classified them into 12 subgroups based on the presence of coiled-coil (CC), nucleotide binding site (NBS), leucine rich repeat (LRR), resistance to Powdery mildew 8 (RPW8), and BED type zinc finger domains. Traditionally classified CC-NBS-LRR (CNL) genes were nested into four clades (CNL A-D) often with abundant, well-supported homogeneous subclades of Type-II R genes. CNL-D members were absent in rice, indicating a unique R gene retention pattern in the rice genome. Genomes from *Arabidopsis*, common bean, poplar and soybean had one chromosome without any CNL R genes. *Medicago* and *Arabidopsis* had the highest and lowest number of gene clusters, respectively. Gene expression analyses suggested unique patterns of expression for each of the CNL clades. Differential gene expression patterns of the nTNL genes were often found to correlate with number of introns and GC content, suggesting structural and functional divergence.

## 1. Introduction

Plants have evolved two types of defense response to pathogen attacks [1]: the first defense is an active response triggered by transmembrane pathogen-associated molecular pattern (PAMP) recognition receptors, also known as PAMP triggered immunity (PTI). This response acts against slowly evolving protein motifs often shared among several types of pathogens [2]. The second type is an effector triggered immunity (ETI) leading to a hypersensitive response (HR) to the pathogen-produced Avirulence (Avr) factors that interrupt signaling of the PTI or modify the cell’s internal environment making it more inhabitable for pathogen infection and growth [3]. The genetic interaction between a plant and its pathogen was first described by Harold Flor [4] as a “Gene-for-Gene Model”, where a plant has a specific Resistance gene (R gene) to defend against a pathogen Avr protein. An alternative to the “Gene-for-Gene Model” is “Guard Model”, which describes interactions of R proteins with Avr targets, rather than direct R-Avr interaction [5], as exemplified by the interaction of RPM1 (Resistance to *Pseudomonas syringae* pv *maculicola* 1) and RPS2 (Resistance to *P. syringae* 2) in *Arabidopsis* [6,7]. In this example, RPM1 is induced to signal when RPM1- interacting protein 4 (RIN4) is phosphorylated by AvrRpm1 and AvrB while RPS2 is triggered as a result of AvrRpt2 degradation of RIN4 in *Arabidopsis* [8,9]. The majority of pathogens are haploid and have shorter generation times, allowing them to evolve faster than their host plants [10]. To cope with the rapid evolution of the Avr molecules, plants have developed rapidly evolving R genes diversified through gene duplication, unequal crossing over, ectopic recombination, gene conversion, and diversifying selection [11,12,13].

Of the major groups of R genes, two groups containing nucleotide-binding site, leucine-rich repeat (NBS-LRR) domains are (a) N-terminal Toll interleukin receptor NBS-LRR or TIR-NBS-LRR or TNL genes, and (b) Non-TNL or nTNL genes including coiled-coil, nucleotide-binding site, leucine-rich repeat (CNL) genes, and resistance to Powdery mildew 8 (RPW8) -NBS-LRR or RNL genes [14]. One primary difference in the evolution of these groups is a near elimination of the TNL genes from monocot genomes [15]. The TNL group differs from the non TNL group in motif composition in the NBS region: Kinase-2 motif of the TNL proteins contain tryptophan (W) while that of the CNL proteins contains aspartic acid (D) [16,17,18]. The NBS region of R genes is often called NB-ARC because of the presence of ARC1 (=APAF-1 [Apoptotic protease activation factor 1] R proteins CED-4 [cell death protein-4]) and ARC2 [19] regions that bind with ATP for phosphorylating downstream signaling targets [1]. The LRR region is located at the C-terminus and is involved in protein-ligand interaction either by sensing the Avr directly or by sensing a change to a specific protein within the cell [1].

Although TNL genes are rare in monocot genomes, nTNL genes are present in all plant genomes, making them suitable for evolutionary comparison [20,21]. The nTNL genes, often classified as CNL genes in literature, were previously identified in *Arabidopsis* [19], and in many plants including members of legumes [10,12,22] and grasses [23]. Recently evolutionary processes including gene clustering, duplication, rates of evolution [24] and effects of chromosomal positioning [25] have been studied in some plant species. Increasingly available genomic data from a wide array of species allows us to perform comparative genomics analyses facilitating identification and characterization of any genes of interest. The main objectives of this research were to compare the nTNL R genes across the genomes of *Arabidopsis thaliana* (At), *Glycine max* (Gm), *Medicago truncatula* (Mt), *Phaseolus vulgaris* (Pv), *Populus trichocarpa* (Pt) and *Oryza sativa* (Os) and elucidate their evolutionary divergence.

## 2. Materials and Methods 

### 2.1. Hidden Markov Model (HMM) Profiling and Sequence Identification

Identification of the nTNL genes in the genomes of *Glycine max*, *Medicago truncatula*, *Oryza sativa*, *Phaseolus vulgaris*, and *Populous trichocarpa* (see Table 1) was similar to that in *Arabidopsis* [19] and Angiosperms wide analysis [21], with slight modification in ortholog search parameters specific to nTNL genes. The 52 *Arabidopsis* nTNL protein sequences were obtained from “The *Arabidopsis* Information Resource (TAIR)” site [26], and used as reference sequences. The NBS-LRR protein sequences for other genomes were obtained from Phytozome site. Hidden Markov Model (HMM) [27] searches were performed based on the multiple sequence alignments of the NBS motif of *Arabidopsis* CNL sequences deposited at the NIB-LRRS database. Thus, the *Arabidopsis* NB-ARC model was then used to scan each of the five other genomes. The results from the HMMscan with an e-value less than 0.05 were retained. All retained sequences were analyzed using InterProScan [28], aligned with ClustalW [29], converted to Stockholm file format, and were used in scanning the respective genome as a genome-specific HMM profile. This time, sequences with e-values less than 0.001 were retained, which were rescanned using InterProScan. The resulting sequences were selected as nTNL genes if they met the following three criteria: (1) sequence had an NB-ARC as predicted by Pfam [30] with the InterPro ID: IPR002182, (2) sequence had a predicted LRR region by InterPro ID: IPR001611 (LRR_1 Pfam) InterPro ID: IPR025875 (LRR_4 Pfam) and InterPro ID: IPR000767 (DISEASERSIST prints) and (3) if the sequences met both of the preceding criteria but had an InterPro ID: IPR000157 (TIR) in the predicted region, the sequences were excluded as TNL genes (see [31] for detail). The presence of an NB-ARC with three conserved motifs P-loop, Kinase 2, and GLPL (Glycine-Leucine-Proline-Leucine) was verified through multiple expectation maximization for motif elicitation (MEME) analysis. The sequences lacking any of these three motifs were excluded from further analysis. The NBS sequences containing all three motifs and DDVD sequence within Kinase-2 motif [16,17,18] were confirmed as CNL or nTNL genes. The genes were then classified based on the presence of coil, NBS, LRR (LxxLxxLxx), RPW8, and BED zinc finger domains.

### 2.2. Sequence Alignment, Phylogenetic Analysis and Conserved Motifs Assessment 

The NB-ARC sequences were aligned using MUSCLE integrated in the program MEGA [38]. Sequences were trimmed ~20 amino acids before the P-loop motif and ~20 amino acids after the GLPL motif. The sequence alignment was manually adjusted, and a Model test was performed in MEGA to determine the best-fit evolutionary model. Maximum-likelihood phylogenetic analysis was performed with evolutionary model JTT+G+I and 100 bootstrap replicates. Trees were rooted using *Streptomyces coelicolor* accession (p25941) as in *Arabidopsis* [19]. In addition, phylogenetic relationships of the six species were estimated using chloroplast *rbc*L gene sequences available at NCBI GenBank database site. Maximum Likelihood phylogenetic analysis was performed using T92+G+I model for 1000 bootstrap replicates, and *Amborella trichopoda* was used as outgroup.

MEME analysis was used to visualize conserved motifs in the NB-ARC region of the identified nTNL genes [39]. Default settings were used for motif identification, except that the number of displayed motifs was set to 20. This allowed tracking variation among previously defined four CNL-groups while maintaining the visibility of the essential domains including the P-loop, Kinase 2, and the GLPL domains [16].

### 2.3. Gene Clustering, Structural and Functional Variation

Chromosome sequence data were downloaded from Phytozome and visualized using Geneious version 5.6.5 [40]. Gene clustering was determined using a 200-kb sliding window. Chromosomal coordinates (Table S1) for gene structure analysis were also obtained from Phytozome.net. Genes were visualized using a 5′ to 3′ orientation using the program Fancy Gene [41]. Genome wide/chromosomal syntenic maps as well as syntenic maps of only nTNL genes were generated using the program SyMAP 4.2 [42], highlighting the chromosomes with highest number of gene clusters in each of the six species. Average K_a_/K_s_ ratios for each subclade were determined using coding sequences.

Gene expression data are available for *Arabidopsis*, *Glycine*, *Medicago*, *Oryza*, and *Populus* at the PlexDB site [43] and are not uniform in their experimental design. Only data with common themes such as time after inoculation between 12 and 72 h were selected, and each plant was challenged by at least one bacterial or viral effector (PlexDB experiments: At 49 [44], Gm 36 [45], Mt 17 [46], Os 3 NCBI GEO accession GSE 16793, Pt 47 NCBI GEO accession GSE23726). A basic local alignment search tool (BLAST) search was used to find the best matches between the NBS-LRR genes and the probes [47]. Each probe was searched only once to reduce redundancy, and 100% probe identity match was used in our analysis. Biological replicates in a given group of data were summarized as average values. The expression profiles were log-2 transformed and heatmaps were created using the program Mayday 2.30 [48].

## 3. Results

### 3.1. Diversity and Phylogenetic Relationship of the Identified nTNL Genes

**Figure 1:**
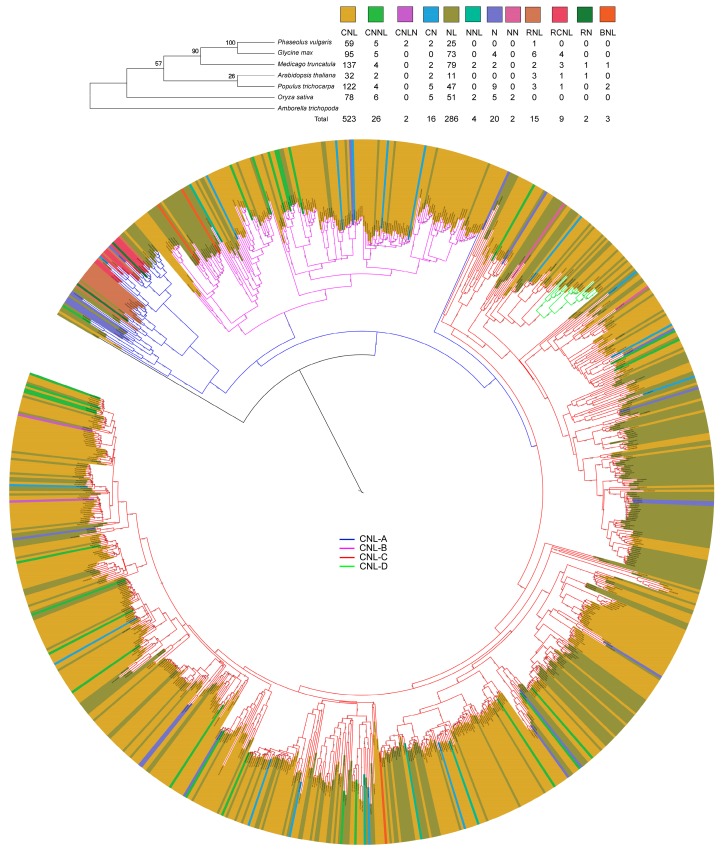
Maximum-likelihood tree of the six plant species (*A. thaliana O. sativa*, *M. truncatula*, *P. vulgaris*, *P. trichocarpa*, *and G. max*) based on *rbc*L gene sequences: the values on the branches represent bootstrap support of 1000 replicates (**upper left**). Diversity of nTNL genes across the genomes from six species: classification of the 12 subgroups was based on the presence of coil, NBS, LRR (Lxx), RPW8, and BED zinc finger domain (**upper right**). Maximum-likelihood tree of the identified nTNL NBS-LRR proteins from six plant species; on the branches are the bootstrap support of 100 replicates (lower middle). Altogether 908 protein sequences were included, using the *Streptomyces* outgroup P25941 (GI: 19857619). Tree branches are color-coded: blue, pink, red, and green for CNL-A, CNL-B, CNL-C, and CNL-D, respectively. The phylogenetic tree was visualized using Interactive tree of life (iTOL) v3. The nTNL gene accessions are color-coded creating a band of 12 colors, exterior to the gene accession names. Branch lengths of 11 sequences (LOC_Os11g42090, LOC_Os11g42070, LOC_Os04g41370, Medtr5g028420, Medtr5g028750, Medtr5g028290, Medtr5g028340, Glyma20g33531, Glyma20g33740, AT5G66910, and AT5G66900) were collapsed to their respective clades to accommodate band size around the tree.

In this study, we confirmed identification of 908 nTNL genes including 52, 187, 233, 149, 94, and 193 from *Arabidopsis*, soybean, *Medicago*, rice, common bean, and poplar, respectively (Table 2). We classified these nTNL genes into 12 categories based on the presence or absence of domains in the protein sequences (see upper right panel; Figure 1). These nTNL (=CNL) genes were nested into four clades: CNL-A, CNL-B, CNL-C and CNL-D (see Figure 1). All four clades were present in dicot species while clade D members were absent in the rice genome. Within-clade distribution of the CNL gene members is shown in Table 2.

Although clade support for CNL-A was weak, each of the CNL-A subclades had a strong bootstrap support (i.e., BS > 90%), and this clade was absent in rice. Clade CNL-A was divided into three subclades: out of which CNL-A2 and CNL-A3 in *Arabidopsis* had prominent orthologs such as activated disease resistance 1 (ADR1) and N requirement gene 1 (NRG1), respectively, however all 26 RNL (RN, RNL and RCNL) members were nested together forming the third subclade (Figure S1, Table S2). Clade CNL-B was moderately supported (BS = 75%) and divided into three subclades. CNL-B1 contained many orthologs of RPS5 and RPS2. RPS5 orthologs in *Arabidopsis* and poplar appeared to have rapidly evolved (Figure S2), while accessions in *Glycine*, *Medicago*, and *Phaseolus* appeared less rapidly evolving. CNL-B2 and CNL-B3 also contained rapidly evolving genes. Clade CNL-C was divided into 13 subclades many of which with poor basal bootstrap support. Some of the well-supported subclades were CNL-C7 (BS = 87%), CNL-C8 (BS = 100%), CNL-C9 (BS = 57%), and CNL-C12 (BS = 65%). Evolutionarily, CNL-C2 and CNL-C12 subclades were more expanded in rice than in other genomes (Figure S3). CNL-D clade was strongly supported (BS = 100%) and lacked rice orthologs. The gene members in this clade were the orthologs of RPP8 or RPP13. The RPP8 gene members were more diversified and rapidly evolving in *Arabidopsis* than in the other species (Figure S4). MEME analysis of amino acid sequence showed that the CNL genes were divergent in their NBS region (Figures S5–S9). The gene members in all species varied in amino acid composition in the region between the P-loop, shown in light blue, and the Kinase-2 motif, shown in red.

**Figure 2:**
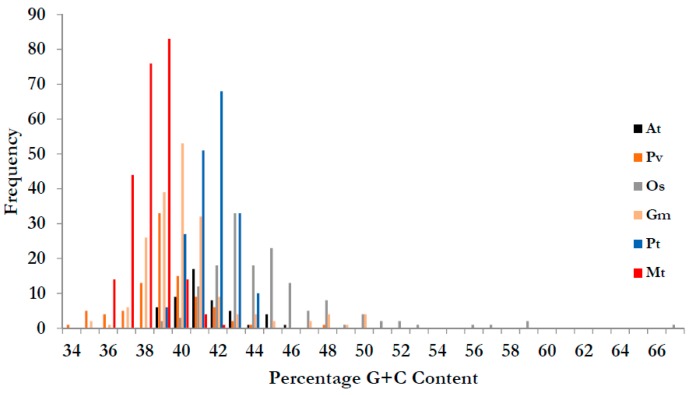
G+C content of the nTNL genes in non-legumes appeared higher than legume species. Rice nTNl genes had highest G+C content among the six species. Species are color-coded: legumes are in red-themed colors (Mt = red, Gm = pink and Pv = dark red) and non-legumes are in non-red colors (At = Black, Pt = Blue, Os = green).

Figure 2 shows average G+C content by genome. Average G+C content of the CNL genes was lower for legumes (*Medicago*, *Glycine* and *Phaseolus*) than for non-legumes (*Arabidopsis*, *Populus* and *Oryza*) with the highest value for rice (monocot) genome. Gene members of clade CNL-A had greater average G+C content in all species than three other clades (CNL-B, CNL-C, and CNL-D).

Exon/intron structure varied across the CNL clades: clade A had the highest average number of exons per sequence in *Arabidopsis*, *Glycine*, and *Populus* with 5.6, 5.4, and 4.6 exons per sequence, respectively; clade B had the highest average number of exons per sequence in *Medicago* and *Phaseolus* with 6.3 and 7.2, respectively. Rice had the highest number of exons per sequences (2.1) in clade C. The structural information is summarized in Table 3. Overall, CNL-A gene members had the most exons per sequence (5.3), followed by CNL-B (4.9), CNL-D (3.8), and CNL-C (2.6). However, the large expansion of CNL-C and the high numbers of *Arabidopsis* sequences in CNL-B and CNL-D may skew this data. The standard deviations for the calculated averages were 1.4, 3.5, 1.8, and 1.3 for CNL-A, B, C and CNL-D, respectively, indicating that CNL-B showed a wider range of variation in exon number while CNL-D had the least variation.

### 3.2. Chromosomal Distribution of nTNL Genes, Gene Clustering and Selection Pressure

Gene clustering analysis showed that the majority of the nTNL genes occurred in clusters in each of the six genomes (Table 4). Four of the six genomes (At, Gm, Pv, and Pt) had one chromosome without nTNL R genes (2, 10, 5, and 10, respectively), while other chromosomes had one or more nTNL members present (Figures S10–S14). The *Medicago* genome contained the most gene clusters (i.e., 35) while the least number of gene clusters were in *Arabidopsis* (i.e., 10). Interestingly, *P. vulgaris* had fewer genes (i.e., 94) relative to other genomes in this study, but had the highest genes per cluster, 5.4, which is heavily influenced by the clusters found on chromosome 11, with 26 clusters.

Selection pressure was surveyed using the ratio of non-synonymous substitution per non-synonymous site (K_a_) to synonymous substitution per synonymous site (K_s_). This survey involved coding sequences of the genes from one subclade of each of the four CNL clades (CNL-A2, CNL-B3, CNL-C13, and CNL-D) (Table 5). The K_a_/K_s_ ratios were less than 1 for CNL-A3, CNL-B3, and CNL-C13 with 14, 32 and 23 members, respectively. The ratios for CNL-D (with 17 members) were below 1 with the smallest being 0.76.

Due to the large number of chromosomes considered in this analysis, generated syntenic maps were limited to chromosomes with highest number of nTNLgene clusters. Chromosomes were named using the first letters of the genus and species name, followed by the chromosome number (i.e., Os11 for *O. sativa* chromosome 11). In order to display synteny regarding the most R gene-dense areas, Figure S15 shows synteny between the chromosomes most populated with R genes and R gene clusters. Specifically focusing on the Os11, which holds a large portion of rice R genes, Figure S16 shows the few cases of synteny between Os11 and the five dicots genomes. As an example of similarities between whole genomes, Figure S17 shows synteny between the *Mediacgo* chromosome 3 (with clusters of nTNL genes) with *Phaseolus* chromosome 5 with high chromosomal synteny but no synteny at the nTNL gene level.

### 3.3. Gene Expression Analysis

Gene expression analysis showed that CNL-A gene members had higher gene expression values than the gene members in other clades (Figure S18). Within the clade CNL-A, 12 of the 30 CNL genes were more highly expressed (Glyma01g39000, Medtr1g021100, Medtr1g021110, Medtr5g018060, Medtr5g018210, Medtr5g036240, Medtr8g079280, Medtr8g079350, Medtr8g079360, Potri.002G129300, Potri.007G039000, and Potri.014G035700). More highly expressed CNL-B gene members included At4G10780, At1G12280, At1G63350, At1G61180, At1G62630, Glyma01g10254, Glyma07g06914, Glyma14g01231, Glyma18g46050, Glyma18g51533, Medtr4g091380, and Potri.006G147100 (Figure S18). Interestingly, the CNL-B members in *Arabidopsis RPS5* (At1g12220) and *RPS2* (At4g26090) were not among the highly expressed genes. The CNL-C clade had 70 highly expressed members (see Table 6 and Figure S19) while CNLD had only two highly expressed genes (At1G10920 and Potri.018G138500) (see Figure S18).

Six of the CNL-A genes (At5G47280, Glyma11g06260, Medtr2g083510, Medtr6g084360, Medtr8g018040 and Potri.013G097200), seven of CNL-B (At5G47260, Medtr5g036460, Potri.001G429700, Potri.001G434000, Potri.001G443900, Potri.001G444000, and Potri.019G002800), 37 members of the CNL-C and one member (AT1G58390) of the CNL-D clade had basal level of expression values (see Table 6). Expression profiles are visualized in Figures S20–S24.

## 4. Discussion 

### 4.1. Phylogenetic History and Motif Structure of the nTNL Genes

Phylogenetic analysis revealed support for both recent rapid evolution of CNL R genes [49] as well as more conserved evolutionary relationships of the selected plant species [50,51,52]. The recent rapid evolution can come in many forms: gene duplication, unequal crossing over, ectopic recombination, gene conversion, and diversifying selection [1]. The orthologs of RPS5 and RPS2 genes that respond to different upstream effectors produced by *P. syringae* [53] form weakly supported sister groups with several paralogs within each group (see Figure S2), possibly formed through recombination events in the leucine rich regions that respond to the effectors. Strong orthology among some gene members showed ancient evolutionary relationships even among distantly related species (Figure 1 and Figure S3). Within clade CNL-C, many separate lineages each with orthologs from multiple species diversified as in subclades CNL-C1, 3, 4, 9 and CNL C13) and perhaps diversified again as in subclade CNL-C 10–12. Ortholgs of CNL-D gene members were absent in rice genome (Figures S1 and S4). Previous study [54] has shown that some CNL-A genes, such as CNL-A3 (*NRG1*) members, function in a pathway that require a TNL gene. All RNL genes were nested forming a subclade of 26 genes (see Table S2) within the clade CNL-A. This subclade includes RNL, RN and RCNL genes, possessing an N-terminal RPW8 domain [55]. Since the TNL type of R genes are absent in monocot genomes [55,56,57,58,59], lack of the CNL-A orthologs in rice indicated its potential dependence on the TNL type R genes. A previous analysis has linked expansion of CNL and TNL genes to the Cretaceous-Paleogene boundary and, similar to our analysis, fewer RNL genes were identified across diverse plant groups [21]. The lack of CNL-D, containing mostly RPP8 and RPP13 orthologs providing resistance to *Hyaloperonospora arabidopsidis* [60], could possibly come from functional independence of RPP8 [61] and RPP13 [62] from enhanced disease suscpetibility 1 (*EDS1*) and non-race specific disease resistance 1 (*NDR1*) pathways that commonly associated with the CNL and TNL type disease resistance genes, respectively.

Based on evolutionary rates, some previous studies have classified CNL resistance genes into two groups: those that rapidly evolve through frequent recombination and sequence exchanges (Type I) and those that evolve more slowly due to infrequent sequence exchange (Type II) [49,63]. Phylogenetic analysis in the present study revealed the presence of both type I and type II genes in each of the six genomes. Rapidly evolving genes were easily identifiable based on the number of paralogs (e.g., CNL-C9 in soybean and CNL-C2 in poplar) or branch lengths (e.g., several CNL-C subclades of soybean and rice). Poplar, only tree species among the six species, displayed relatively shorter branch lengths compared to the herbaceous species (Figure S3), perhaps because of poplar’s woody habit [64] (i.e., trees having longer generation time than herbs). As shown in Figure S4, rate of evolution of the CNL-D genes is slower, indicated by lower diversification of the CNL genes although soybean RPP8 orthologs of *Arabidopsis* are highly diversified. A previous study has classified rice R genes as Type II, with high sequence conservation [23]. Figure S4 presents an example of Type II genes, where soybean *RPP8* is nested with its orthologs in *Phaseolus*, which are in turn nested with *Medicago* ortholog and *RPP13* gene of poplar. During the identification process, pseudogene sequences were removed if a stop codon was found prior to the LRR regions. The nTNL genes identified in this study had these conserved domains: P-loop, Kinase-2, and GLPL, typically containing conserved motifs (P-loop, RNBS-A, Kinase-2, RNBS-B, RNBS-C, and GLPL). Lesser conserved regions before and after the Kinase-2 domain are useful in predicting CNL-subgroups [19]. Similar to our findings, previous studies have shown that Kinase-2 motif possess DDVD and DDVW amino acid residues in TNL and non-TNL proteins, respectively [16,17,18]. In common bean the P-loop directly could be used to separate between the CNL-A or CNL-B ancestry and that of CNL-C or CNL-D ancestry. Similar to our results, non-TNL genes have been shown to possess diverse splicing patterns, with many genes having no introns [65].

### 4.2. Chromosomal Distribution of nTNL Gene Clustering

A comparison among NBS encoding genes from *A. thaliana*, *Brassica rapa* and *Brassica oleracea* suggested species specific gene diversification patterns in the two *Brassica* species [66], indicating even high degree of divergence among distantly related species used in the present study. This study compared the nTNL genes from one monocot species (Os) and five dicot species (three legumes [Gm, Mt and Pv], one non-legume representing Rosid I [Pt], one representing Rosid II [At]). Selection pressure, surveyed using the ratio of non-synonymous (K_a_) to synonymous (K_s_) substitution within the coding region, was estimated for one subclade from each of the CNL clades CNL-A2, CNL-B3, CNL-13C, and CNL-D (no subclades) (Table 5). Clade CNL-A, CNL-B, and CNL-C with 14, 32, and 23 members, respectively, had K_a_/K_s_ ratios below one, suggesting purifying selection. This was in contrast to CNL-D (17 members) largely demonstrating diversifying selection (K_a_/K_s_ > 1). Out of the three inferred cases of purifying selection in this clade, the smallest K_a_/K_s_ ratio was 0.76 (between Glyma15g18290 and Phavul.009g233700). The majority of the gene members identified in this study were in the CNL-C clade. In each of the four CNL clades were clusters of gene members evolved through tandem duplications (e.g., five-membered cluster on chromosome 1 [all in clade CNLD: At1G58390, At1G58400, At1G58410, At1G59124, and At1G59218]; Figure S10). Pairwise comparison of K_s_ values among these five accessions presented evidence of tandem duplication consistent to the findings in soybean [12], the pairwise K_s_ values increased as the sequence location gets farther away from At1G59124 through At1G58390.

Centromeric positioning of disease resistance genes is likely to influence their ability to duplicate and transcribe. Pericentromeric regions have been linked for influencing gene retention, expression, and duplication in soybean [25] and in common bean [67]. Each of the 15 clusters of the common bean genome occurs outside the predicted pericentromeric region. Although the pericentromeric regions of *Medicago* chromosomes have yet to be defined, visual analysis of the physical clustering of nTNL genes shows it might have little or no effect in some cases in *Medicago*. For example, *Medicago* chromosome 1 and 8 have distantly located nTNL gene clusters from the centromere, but on chromosome 3 the genes and clusters appear very close to the centromere itself. Rice nTNL genes are not as densely clustered as in *Medicago*, but the clusters are located both near to and far away from the centromere. In *Arabidopsis* the NBS-LRR genes tend to cluster away from the centromere, possibly allowing these genes to evolve through recombination.

Focusing on the chromosomes with the largest number of nTNL genes and their clusters presented in this paper, we found high synteny at the genome level among or between the species but the majority of the nTNL genes had very little synteny at the gene level across the six genomes. Especially larger clusters were found in common bean, poplar, and *Medicago*, with 26, 18, and 15 genes, respectively. *Medicago* chromosome three (Mt03) has twice as many R genes in it than any single chromosome of the six species. The nTNL-dense Mt03 shows synteny with chromosomes in each of the other five species, with large blocks of synteny in common bean and soybean, and smaller, more fragmented blocks of synteny in the *Arabidopsis*, poplar, and rice. In addition to Mt03, the next most populated chromosomes with 20 or more R genes, from the highest to the lowest, are Mt05, Pt01, Os11, Gm18, Pv11, Pt17, At01, and Gm3. Syntenic map comparing these nine chromosomes reveals that all exhibit some similarity at varying levels (Figure S15). At01 displays similarity at many locations within the other chromosomes, except Os11. Rice Os11 contains a large portion of its nTNL genes (approximately 27%). A syntenic map using Os11 as a reference shows that many of the nTNL genes on this chromosome are less closely related to the regions in the five other genomes indicating potential diversification of the largest gene clusters within monocot genome after the dicot-monocot split (Figure S16). No syntenic blocks were seen between rice and *Arabidopsis*, and only a few small sections were found in common bean, *Medicago*, and poplar. The only major exception is a segment of Gm19 that has a large syntenic block with Os11. This lack of dicot synteny with Os11 does not exist when comparing rice to other monocot genomes, as exemplified by the high similarity between Os11 and foxtail millet chromosome 9 (Si09) [18]. This would indicate that the large expansions responsible for this clustering in rice might have occurred after the monocot-dicot divergence, and soybean may have had an independent expansion due to polyploidization events. In addition, a large population of nTNL gene clusters on the short arm of Mt03, provides an evidence nTNL gene diversification through tandem duplication. This region shows major blocks of synteny with Pv05 (Figure S17), which, however does not contain nTNL genes. The massive amount of genes from this region also generally nest together in large clades (see Figure 1), indicating that they arose from duplication after the ancestor of *Medicago* had diverged from the other species.

Overall, comparison of chromosomal syntenic maps with the synteny at the gene level suggested that the chromosomal syntenic maps furnish general patterns of evolution at the genome or chromosome level (across these distantly related species), however, it may not serve as evidence to support our reasoning of nTNL gene diversification. It is in part due to evolution of the majority of the nTNL genes through tandem duplications (see new Figure S17).

### 4.3. Assessing Gene Expression Data 

Expression values of the CNL-A gene members were high in all species as shown in Figure S18. Relative to three other clades, CNL-A had a disproportionate number (12 of the 30) of highly expressed genes in three species (Glyma01g39000, Potri.002G129300, Potri.007G039000, Potri.014G035700, Medtr1g021100, Medtr1g021110, Medtr5g018060, Medtr5g018210, Medtr5g036240, Medtr8g079280, Medtr8g079350 and Medtr8g079360), data not available for *P. vulgaris*. The expression values were below average for the CNL-A orthologs in *Arabidopsis*, and the CNL-A clade was absent in rice. CNL-A clade had the highest expression values in *Arabidopsis*, *Medicago*, and poplar, but second highest in soybean. CNL-B had 12 highly expressed genes (Glyma01g10254, Glyma07g06914, Glyma14g01231, Glyma18g46050, Glyma18g51533, Potri.006G147100, Medtr4g091380, At4G10780, At1G12280, At1G63350, At1G61180, and At1G62630) (Figure S18). The CNL-B orthologs did not have an as easily discernible expression patterns as the CNL-A genes had in terms of gene homology associated with specific functions. The only gene homologies in this clade were RPS5 (At1g12220) and RPS2 (At4g26090), which did not appear to be among the highly expressed genes. The proportion of highly expressed genes in clade CNL-C corresponded to the clade size (Figure S19), with 70 highly expressed sequences (see Table 6), while clade CNL-D had only two highly expressed genes (Potri.018G138500 and At1G10920; see Figure S18). CNL-A genes were nested into three subclades, two of which were similar to a previous study by Collier (2011) [55]. The highly expressed sequences could represent those genes that are necessary for pathways other than the disease signaling. The clade of Activated Disease Resistance 1-Like 1 (*ADR1-L1*; AT4G33300) orthologs does not contain a member from common bean, while the clade of *NRG1* (N requirement gene 1) orthologs includes one member from common bean genome. Both *NRG1* and *ADR1* proteins are possible downstream targets in the defense signaling pathway, which could explain why the CNL-A group contains proportionally higher number of highly expressed genes [54].

In apple, physical association of R genes and expression values were used in inferring functional relationships [68]. The expression proximity of these genes did not provide enough information to predict the gene function. In the case of the genes that were associated with powdery mildew (PM) resistance that were physically clustered had expression levels that were positively associated with the phenotype while other genes although physically clustered were negatively associated the PM-resistant phenotype. This method may not be a good way of predicting the expression levels but it does seem to have a likelihood of relating to functionality of the gene. The expression profiles from our study showed tendencies similar to those suggested in Chaudhary et al. 2008 [69], where domesticated plants had more down-regulated genes compared to the wild species. The differences in the nTNL gene regulations are expected, as these species have differing domestication history. In the species with longer history of domestication, selection pressure among these genes is relatively weak restricting their diversification. In the CNL-A 1 (NRG1) clade of *Medicago*, there are several highly expressed members (Medtr5g018210, Medtr5g018060, Medtr8g079280, Medtr8g07350, and Medtr8g079360, with much fewer highly expressed orthologs in other genomes. Among other factors influencing the diversification of R genes, tandem and whole genome duplications constitute major forces. Recently, tandem duplications was found more common than whole genome duplication in generation of NBS-LRR genes in *Arabidopsis* and *Brassica* species [66].

The results from this study showed that nTNL genes from dicot genomes had higher G+C content than that from the monocot genome, consistent to previous findings [70]. Among the dicot genomes analyzed in this study, CNL-A gene members had higher than other CNL clades. Interestingly, these gene members had also higher gene expression values. These findings are consistent with previous findings describing a correlation between expression values and the G+C content [71]. This potential correlation may be attributable to the assumption that increased GC content would increase stability of the DNA strands in the genome, an assumption that warrants further investigation. The results from this study also showed that expression values and number of introns were typically higher for CNL-A and CNL-B gene members compared to that for CNL-C and CNL-D gene members. The results were similar to *Arabidopsis* [19] suggesting a potential intron-mediated expression as described by Rose 2008 [72]. Overall, we presented data showing differential expression of the nTNL genes in the six species that differed in their domestication history [69]. Further characterization of the highly expressed genes would have implications in developing cultivars with durable resistance while the genes with basal level expression, perhaps have already subfunctionalized [73,74], are important reservoirs of genetic diversity.

## 5. Conclusions

In this study, altogether 908 nTNL genes were identified and analyzed from six plant genomes (*A. thaliana*, *G. max*, *M. truncatula*, *O. sativa*, *P. vulgaris* and *P. trichocarpa*). These genes were classified into 12 groups, and nested into four clades. Their evolutionary history indicated their diversification through potential recombination, tandem or genomic duplication events. The duplication events perhaps left physical clusters of the nTNL genes in each genome. Differential gene expression profiles of the nTNL genes often correlated with the number of introns, G+C content and domestication history suggest their functional divergence. Future research on further characterization of these genes would lead to genetic modifications to produce durable resistance in crops.

## Figures and Tables

**Table 1:** Domestication history and genomic information of *Arabidopsis* [32], Soybean [33], Rice [34], *Medicago* [35], Common bean [36] and Poplar [37].

Plant Species	Domestication (Years Ago)	Native Range	Chromosome Number (*N*)	Genome Size
*Arabidopsis thaliana*	None	Eurasia (adaptive elsewhere)	5	135 MB
*Glycine max*	6000–9000	Central or Northern China	20	1100 MB
*Oryza sativa*	11,000–12,000	Southern China	12	383 MB
*Medicago truncatula*	None	Mediterranean basin	8	465 MB
*Phaseolus vulgaris*	4400	Americas (Argentina, Guatemala)	11	650 MB
*Populus trichocarpa*	Literature unclear before 1977	North Western United States ranging from Alaska to California and as far east as Western North Dakota	19	423 MB

**Table 2:** Distribution and diversity of nTNL (non-Toll interleukin receptor, nucleotide-binding site, eucine-rich repeat) genes in six different plant species (At = *Arabidospis thaliana*, Gm = *Glycine max*, Mt = *Medicago truncatula*, Os = *Oryza sativa*, Pv = *Phaseolus vulgaris* and Pt = *Populus trichocarpa).*

Clades	At	Gm	Mt	Os	Pv	Pt	Total
CNL-A	8	14	19	0	1	7	49
CNL-B	25	37	22	3	17	61	165
CNL-C	7	134	191	145	75	124	676
CNL-D	12	2	1	0	1	1	17
Unnested	0	0	0	1	0	0	1
Total	52 ^a^	187	233	149 ^b^	94	193	908

^a^ Three sequences AT4G19050 (NL-A), AT5G66630 (CN-A), and AT3g15700 (CN-B) [19] did not contain either a Leucine-rich region or the coiled-coil region. ^b^ Previously reported CNL member LOC_Os12g10410 in rice was highly divergent and did not nest inside of the four CNL clades.

**Table 3:** Intron-exon structure of the identified nTNL genes in six plant species (*Arabidopsis*, soybean, *Medicago*, rice, common bean and poplar). Max = maximum number of exons, Min = minimum number of exons and Avg. = average number of exons.

Species	CNL-A			CNL-B			CNL-C			CNL-D		
	Max	Min	Avg.	Max	Min	Avg.	Max	Min	Avg.	Max	Min	Avg.
At	10	3	5.6	4	1	1.5	2	1	1.1	6	3	4.3
Gm	7	2	5.2	15	1	7.1	9	1	2.7	4	2	3.0
Mt	7	4	5.4	14	1	6.3	17	1	2.7	2	2	2.0
Os	0	0	0.0	2	1	1.7	8	1	2.6	0	0	0.0
Pt	8	2	4.7	11	1	3.7	10	1	2.4	2	2	2.0
Pv	5	5	5.0	16	2	8	7	1	2.3	2	2	2.0

**Table 4:** Gene clustering in six plant genomes as predicted by 200 kb sliding windows. (At = *A. thaliana*, Gm = *G. max*, Mt = *M. truncatula*, Os = *O. sativa*, Pv = *P. vulgaris*, and Pt = *P. trichocarpa*).

Species	Count of Clusters Observed	Genes in Clusters	Total nTNL Genes in Clusters (%)	Genes in Largest Cluster	Average Genes Per Cluster
At	10	29	58%	5	2.90
Gm	33	134	71%	11	4.06
Mt	35	190	81%	15	5.43
Os	29	74	50%	7	2.64
Pv	15	82	87%	26	5.47
Pt	34	156	80%	18	4.59

**Table 5:** Summary of K_a_/K_s_ ratios for the nTNL gene members from CNL-A2, CNL-B3, CNL-C13 and CNL-D groups.

Sub Clade (Number of Gene Members)	Minimum K_a_/K_s_	Maximum K_a_/K_s_	Average K_a_/K_s_
CNL-A2 (14)	0.09	0.43	0.20
CNL-B3 (32)	0.10	0.81	0.27
CNL-C13 (23)	0.12	0.55	0.30
CNL-D (17)	0.76	2.29	1.55

**Table 6:** CNL-C genes from the genomes of five different species showed differential gene expression (see Figures S20–S24 for more information).

Highly expressed Genes	AT3G46710, AT3G14470, Glyma20g33740, Glyma09g02401, Glyma12g14700, Potri.001G025400, LOC_Os01g23380, LOC_Os01g25720, LOC_Os01g33684, LOC_Os01g57270, LOC_Os01g57280, LOC_Os02g16270, LOC_Os02g17304, LOC_Os02g25900, LOC_Os03g40194, LOC_Os03g63150, LOC_Os04g02110, LOC_Os05g30220, LOC_Os05g31570, LOC_Os05g34220, LOC_Os05g34230, LOC_Os07g17250, LOC_Os07g29820, LOC_Os08g07330, LOC_Os08g10260, LOC_Os08g31800, LOC_Os08g43010, LOC_Os10g10360, LOC_Os11g12000, LOC_Os11g12340, LOC_Os11g37740, LOC_Os11g37759, LOC_Os11g42070, LOC_Os11g44580, LOC_Os11g44960, LOC_Os11g45790, LOC_Os12g32660, LOC_Os12g33160. LOC_Os12g37760. Medtr1g016210, Medtr2g014820, Medtr3g014080, Medtr3g015260, Medtr3g027420, Medtr3g032150, Medtr3g055720, Medtr3g056190, Medtr3g056300, Medtr3g070590, Medtr5g021080, Medtr6g046930, Medtr6g047210, Medtr6g052390, Medtr7g089080, Medtr7g091110, Medtr8g011280, Medtr8g038590Potri.001G134700, Potri.001G261300, Potri.003G149800, Potri.003G201800, Potri.005G119800, Potri.006G014400, Potri.006G271800, Potri.007G137100, Potri.008G212200, Potri.011G040800, Potri.012G121900, Potri.017G121500, and Potri.017G136400
Genes expressed at the basal level	Glyma01g35120, Glyma02g03010, Glyma02g03520, Glyma03g05772, Glyma05g08621, Glyma13g26000, Glyma13g26141, Glyma13g26380, LOC_Os01g25630, LOC_Os03g36920, LOC_Os03g50150, LOC_Os05g12770, LOC_Os05g31550, LOC_Os05g40150, LOC_Os09g13820, LOC_Os10g03570, LOC_Os10g07400, LOC_Os11g45160, LOC_Os11g45970, LOC_Os12g29280 and LOC_Os12g31620, Medtr1g023600, Medtr2g039010, Medtr3g035480, Medtr3g086070, Medtr5g035240, Medtr5g070470, Medtr6g046440, Medtr8g011590, Medtr8g011600, Potri.003G099000, Potri.004G195200, Potri.012G122200, Potri.017G127000, Potri.017G143400, Potri.017G143500, and Potri.018G017900

## Supplementary Materials

The following are available online at www.mdpi.com/2073-4425/8/10/249/s1. Figure S1: CNL-A clade with gene members nested into three subclades, Figure S2: CNL-B clade with gene members nested into three subclades, Figure S3: CNL-C clade with gene members nested into 13 subclades, Figure S4: CNL-D clade with gene members, including RPP8 and RPP13 orthologs, Figure S5: MEME analysis and predicted domain structure of nTNL genes in *A. thaliana* CNL. Conserved domain structures as predicted by MEME analysis of CNL genes in *Arabidopsis*. Sequences are grouped by CNL subgroup. The first three sequences appearing at the top (AT4G1905 (NL-A), AT5G66630 (CN-A), AT3g15700 (CN-B)) are without CNL designation. On the right are shown twenty conserved domains searched and weblogo visualization of residue frequency, Figure S6A–C: MEME analysis predicted domain structure of *M. truncatula* nTNL genes. Twenty different sites were analyzed for prediction of conserved domain structures. Sequences are grouped by CNL subgroup. (A) Contains the motif WebLogo and legend along with sequences belonging to CNL-A, CNL-B and CNL-D. (B) Contains sequences that belong to CNL-C that appearing on chromosome 1 through chromosome 4. (C) contains sequences that from Clad CNL-C appearing on chromosome 5 through Chromosome 8, Figure S7A–B: MEME analysis and predicted domain structure of *O. sativa* nTNL genes. Twenty different sites were searched for conserved domain structures. Sequences are grouped by their CNL subgroup. Three CNL-B sequences are located at the top of column one and one sequence that did not lie in any of the other CNL-sub groups identified as unsettled is listed at the bottom of column two. All other sequences belong to CNL-C of rice. (A) Contains the Motif weblogo and legend along with the sequences belonging to CNL-B the single unsettled sequence and CNL-C sequences appearing on chromosome 1 through chromosome 5. (B) Contains CNL-C sequences appearing on chromosome 6 through chromosome 12, Figure S8: MEME analysis and predicted domain structure of *P. vulgaris* nTNL genes. Twenty different sites were searched for conserved domain structures. Sequences are grouped by their CNL subgroup, Figure S9A–B: MEME analysis and predicted domain structure of *P. trichocarpa* nTNL genes. Twenty different sites were searched for conserved domain structures. Sequences are grouped by their CNL subgroup. (A) Contains accessions from CNL-A, B, and D. (B) Contains accessions from CNL-C. Figure S10: Chromosomal distribution of the nTNL genes of *A. thaliana* (2N = 10). Black lines represent chromosomes, black ovals represent centromeres, and arrows indicate the location and orientation of CNL genes. Gene clades CNL-A, CNL-B, CNL-C and CNL-D were color-coded in blue, pink, red and green, respectively. Figure S11: Chromosomal distribution of the nTNL genes of *M. truncatula* (2N = 16). Black lines represent chromosomes, black ovals represent centromeres, and arrows indicate the location and orientation of CNL genes. Gene clades CNL-A, CNL-B, CNL-C and CNL-D were color-coded in blue, pink, red and green, respectively. Figure S12: Chromosomal distribution of the nTNL genes of *O. sativa* (2N = 24). Black lines represent chromosomes, black ovals represent centromeres, and arrows indicate the location and orientation of CNL genes. Gene clades CNL-A, CNL-B, CNL-C and CNL-D were color-coded in blue, pink, red and green, respectively. Figure S13: Chromosomal distribution of the nTNL genes of *P. vulgaris* (2N = 22). Black lines represents chromosomes, black ovals represent centromeres, yellow ovals represent the pericentromeric regions, and arrows indicate the location and orientation of CNL genes. Gene clades CNL-A, CNL-B, CNL-C and CNL-D were color-coded in blue, pink, red and green, respectively. Figure S14: Chromosomal distribution of the nTNL genes of *P. trichocarpa* (2N = 36). Black lines represent chromosomes, black ovals represent centromere, and arrows indicate the location and orientation of CNL genes. Gene clades CNL-A, CNL-B, CNL-C and CNL-D were color-coded in blue, pink, red and green, respectively. Figure S15: Syntenic map of chromosomes with high populations of nTNL genes in the six plant species, Figure S16: Syntenic map of chromsomes showing synteny with chromosome 11 in rice (Os11), Figure S17: Phylogeny of nTNL accessions from the short arm of chromosome Mt03 (left panel), along with the major block of synteny with this region, found on chromosome Pv05 (right panel), Figure S18: Abundance of number of expressed CNL-A, CNL-B, and CNL-D in available plant genomes (At = *A. thaliana*, Gm = *Glycine max*, Mt = *Medicago truncatula*, Os = *Oryza sativa*, and Pt = *Populus trichocarpa*). The expression level are color coded: abundence of CNL gene members with expression level above average are shown in red while those below average are in blue. Figure S19: Abundance of number of expressed CNL-C genes in five plant genomes (At = *A. thaliana*, Gm = *Glycine max*, Mt = *Medicago truncatula*, Os = *Oryza sativa*, and Pt = *Populus trichocarpa*). The expression level are color coded: abundence of CNL gene members with expression level above average are shown in red while those below average are in blue. Figure S20: Gene expression data for *A. thaliana* nTNL gene members are visualized as heatmaps. Log 2-base value was employed to construct heatmaps. Samples were collected 24 h after inoculation. Control was a mock inoculation. Avrpstdc3000, represents data collected from pathogenic leaf bacterium (*P. syringae* pv. *abrassicicola*), was a pathogenic leaf fungus (*Alternaria brassicicola*), *P. rapae* was a tissue-chewing caterpillar (*Pieris rapae*) and F-occidentalis, cell-content-feeding thrips (*Frankliniella occidentalis*). Figure S21: Gene expression data for *G. max* nTNL gene members are visualized as heatmaps. Log 2-base value was employed to construct heatmaps. All samples were collected 24 h after inoculation. Control (Moc) was the mock inoculation data at 24 h. Tw80-2 samples are representations of virulent soybean rust *Phakopsora pachyrhizi* (tw80-2). Hw94-1 represents an avirulent soybean rust challenge (hw94-1) from the same pathogen. Figure S22: Gene expression data for *M. truncatula* nTNL gene members are visualized as heatmaps. All experimental samples were collected 72 h after inoculation (hai). Each of the three conditions had its own control (identified with moc) taken 0 hai. *Ralstonia solanacearum* is the causal agent of the devastating bacterial wilt disease. *Ralstonia solanacearum* was analyzed in the pathosystems A17 (identified as A17 72 hai) and F83005.5 (identified as f83005.5 72hai) resistant *M. truncatula* lines infected with the pathogenic strain GMI1000. The mutant A17 line, Sickle (identified as sickle 72 hai), which showed a resistant phenotype was also part of the experiment. Figure S23: Gene expression data for *O. sativa* nTNL gene members are visualized as heatmaps. All experimental samples were collected at 24 h after inoculation. Xoo represents samples collected from leaves inoculated with Bacterial blight *Xanthomonas oryzae* pathovar (pv.) *oryzae* and Xoc bacterial leaf streak *X. oryzae* pv. *oryzicola.* The control for this experiment was a mock inoculated sample examined 24 h after inoculation. Figure S24: Gene expression data for *P. trichocarpa* nTNL gene members are visualized as heatmaps. Control samples (identified as Moc) were taken at 0 h after inoculation. Experimental samples (MARSSONINABRUNNEAIN 2DAI) were taken 48 h after inoculation of fungal pathogen *Marssonia brunneain.* Table S1: Exon locations within the nTNL genes of the six species. Each gene accession is listed along with its corresponding exon locations, described by chromosome location, nucleotide length within gene, and exon rank, Table S2: Protein domain structure of RNL (RNL, RN, and RCNL) proteins, as annotated in InterProScan. All 26 sequences nested within the CNL-A clade (Figure 1). In terms of statistical significance, all “*Arabidopsis* broad-spectrum mildew resistance protein RPW8” domains had E-values between 2.3 × 10^−7^ and 2.8 × 10^−45^, whereas RPW8 domain profiles were all between 8.6 and and 31.5.
