# Supplementary material for: Comparative Genomics of Non-TNL Disease Resistance Genes from Six Plant Species
Source: Genes (Basel). 2017 Sep 30;8(10):249. doi: 10.3390/genes8100249 (PMC5664099; doi:10.3390/genes8100249)
Supplement: Supplementary file 1 [file genes-08-00249-s001.zip › 8_31_17_SupplementaryDocuments V2/Table S2.docx]

**Table S2.** Protein domain structure of RNL (RNL, RN, and RCNL) proteins, as annotated in InterProScan*.* All 26 sequences nested within the CNL-A clade (Figure 1). In terms of statistical significance, all “Arabidopsis broad-spectrum mildew resistance protein RPW8” domains had E-values between 2.3x10^-7^ and 2.8x10^-45^, whereas RPW8 domain profiles were all between 8.6 and 31.5.

| **Accession** | **Protein Length** | **Arabidopsis broad-spectrum mildew resistance protein RPW8** | **RPW8** | **Coil** |
| --- | --- | --- | --- | --- |
| AT1G33560 | 787 | 12-119 | 1-149 | - |
| AT4G33300 | 816 | 9-130 | 1-149 | - |
| AT5G04720 | 811 | 8-125 | 1-147 | - |
| AT5G66900 | 809 | 10-128 | 1-150 | 55-75, 494-514 |
| AT5G66910 | 815 | 12-133 | 1-150 | - |
| Glyma01g39000 | 822 | 7-110 | 1-146 | 48-75 |
| Glyma01g39010 | 835 | 3-133 | 1-150 | 52-72 |
| Glyma05g09440 | 866 | 53-120 | - | 524-544 |
| Glyma11g06260 | 835 | 2-133 | 1-150 | - |
| Glyma14g08700 | 839 | 29-149 | 17-165 | - |
| Glyma14g08710 | 816 | 9-137 | 1-149 | - |
| Glyma17g20860 | 843 | 28-99 | - | - |
| Glyma17g21360 | 804 | 7-132 | 1-149 | 37-71 |
| Glyma17g36400 | 820 | 9-137 | 1-149 | - |
| Glyma17g36420 | 825 | 11-133 | 1-149 | - |
| Medtr1g021100 | 844 | 9-133 | 1-149 | - |
| Medtr1g021110 | 823 | 12-136 | 1-148 | - |
| Medtr5g018060 | 829 | 4-131 | 1-146 | 62-82 |
| Medtr5g018120 | 805 | 4-133 | - | 62-82 |
| Medtr5g018210 | 836 | 2-133 | 1-150 | 66-86, 101-121 |
| Medtr5g018910 | 615 | 2-122 | 1-140 | - |
| Phvul.001G018800 | 824 | 9-133 | 1-149 | - |
| Potri.002G129300 | 832 | 7-135 | 1-149 | - |
| Potri.007G038700 | 887 | 4-142 | - | 14-41, 201-221 |
| Potri.007G039000 | 837 | 8-143 | 4-154 | - |
| Potri.014G035700 | 834 | 9-138 | 1-149 | - |
