# Supplementary figures and images for: Comparative Genomics of Non-TNL Disease Resistance Genes from Six Plant Species
Source: Genes (Basel). 2017 Sep 30;8(10):249. doi: 10.3390/genes8100249 (PMC5664099; doi:10.3390/genes8100249)

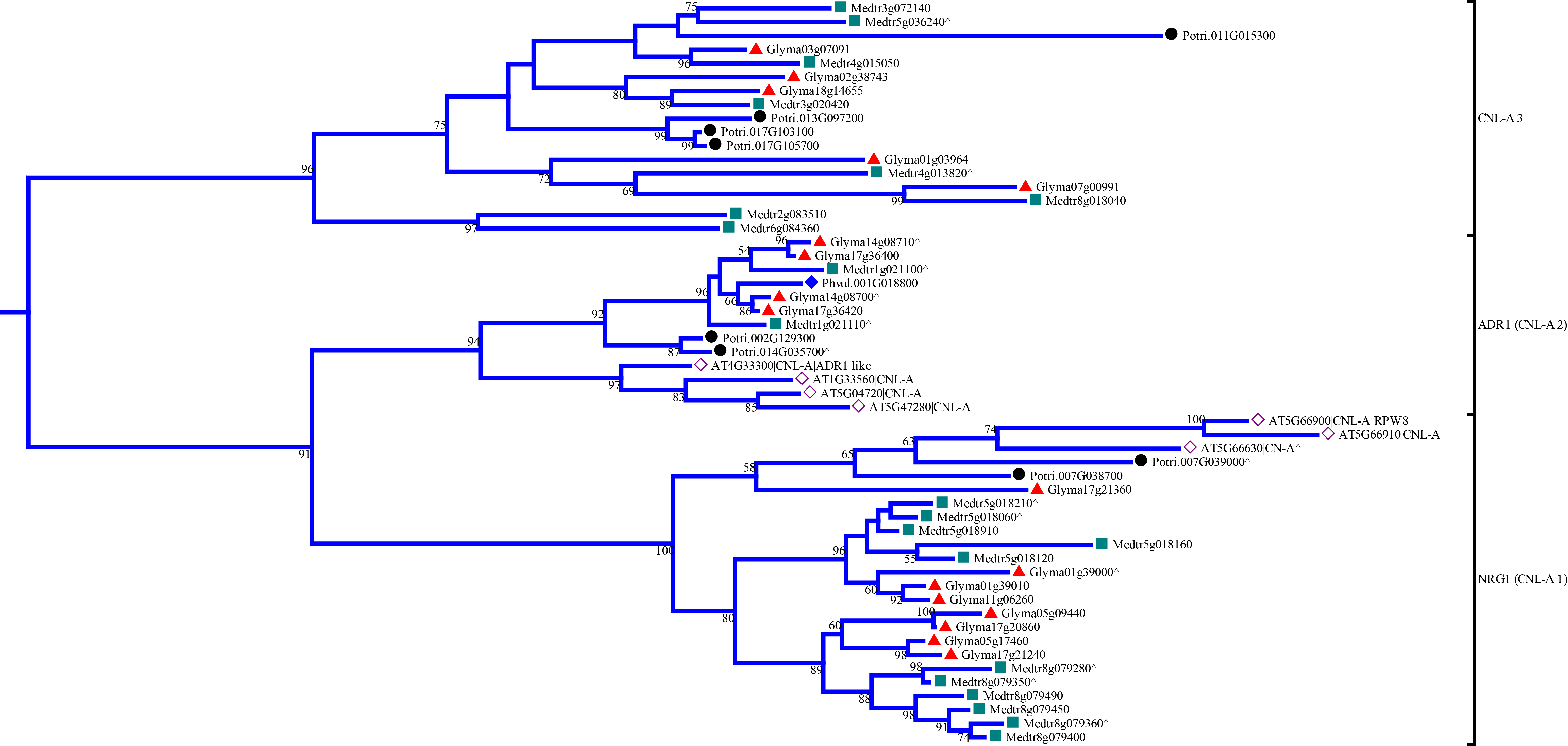

Supplement: Supplementary file 1 [file genes-08-00249-s001.zip › 8_31_17_SupplementaryDocuments V2/Figure S1.tif]

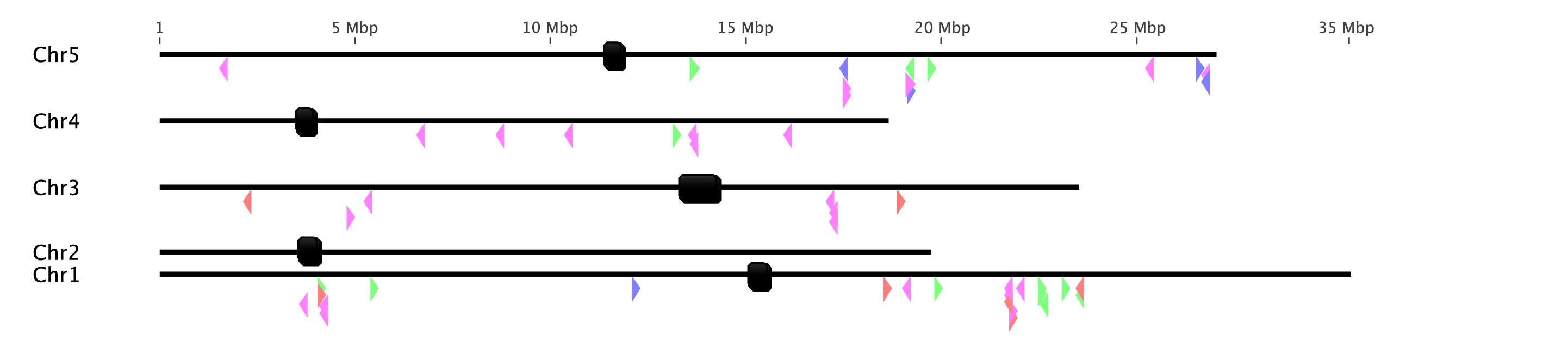

Supplement: Supplementary file 1 [file genes-08-00249-s001.zip › 8_31_17_SupplementaryDocuments V2/Figure S10.tif]

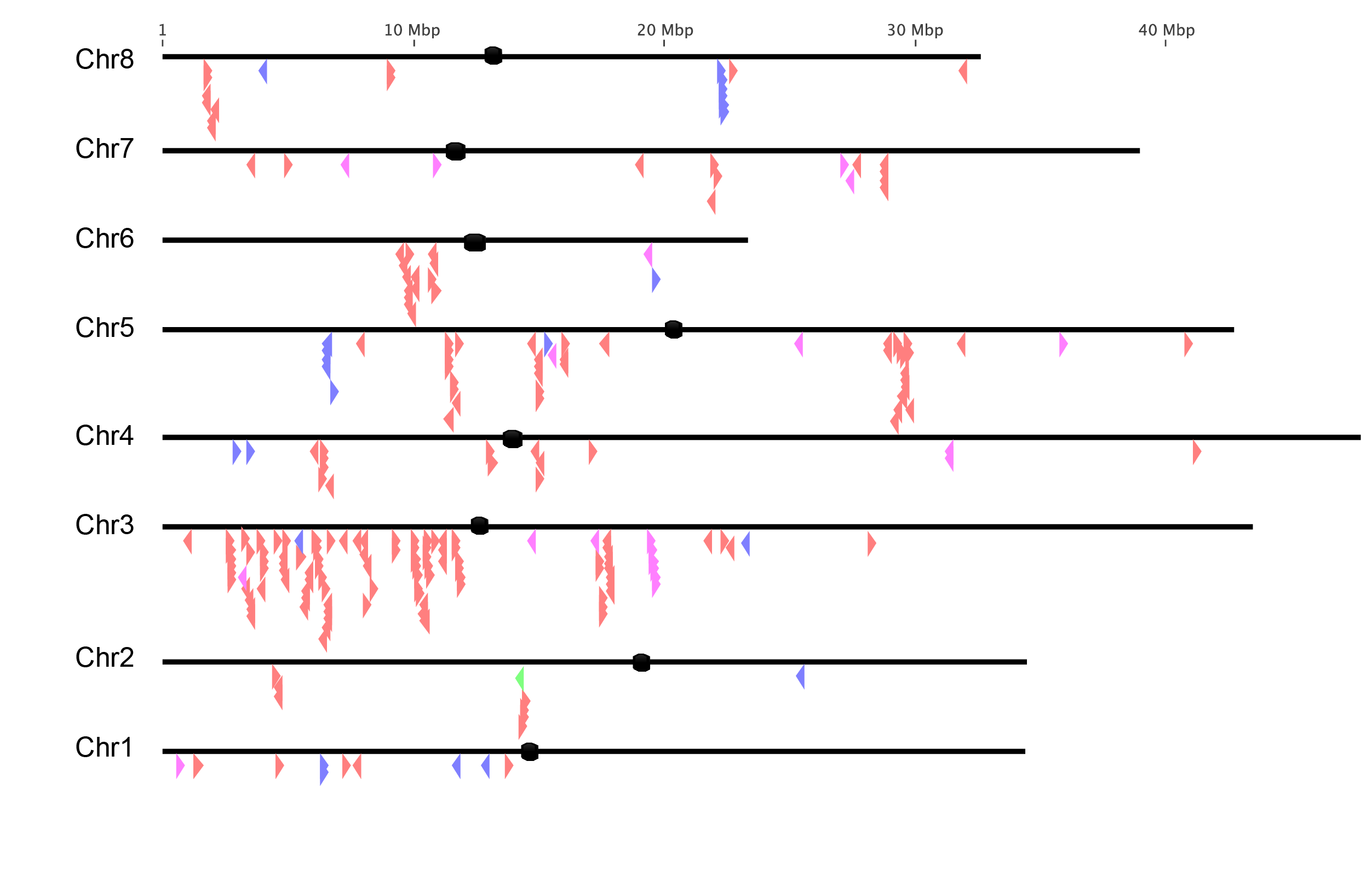

Supplement: Supplementary file 1 [file genes-08-00249-s001.zip › 8_31_17_SupplementaryDocuments V2/Figure S11.tif]

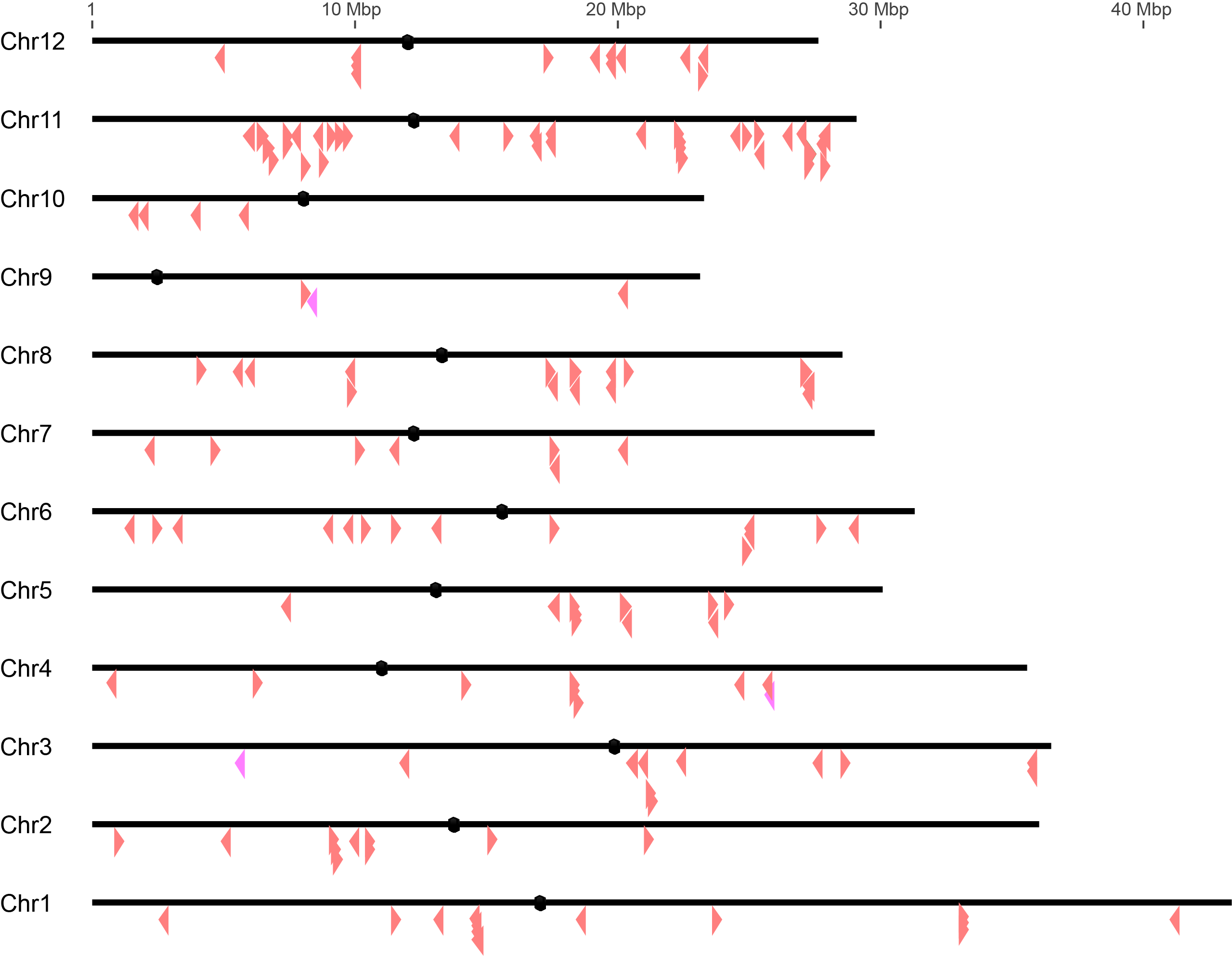

Supplement: Supplementary file 1 [file genes-08-00249-s001.zip › 8_31_17_SupplementaryDocuments V2/Figure S12.tif]

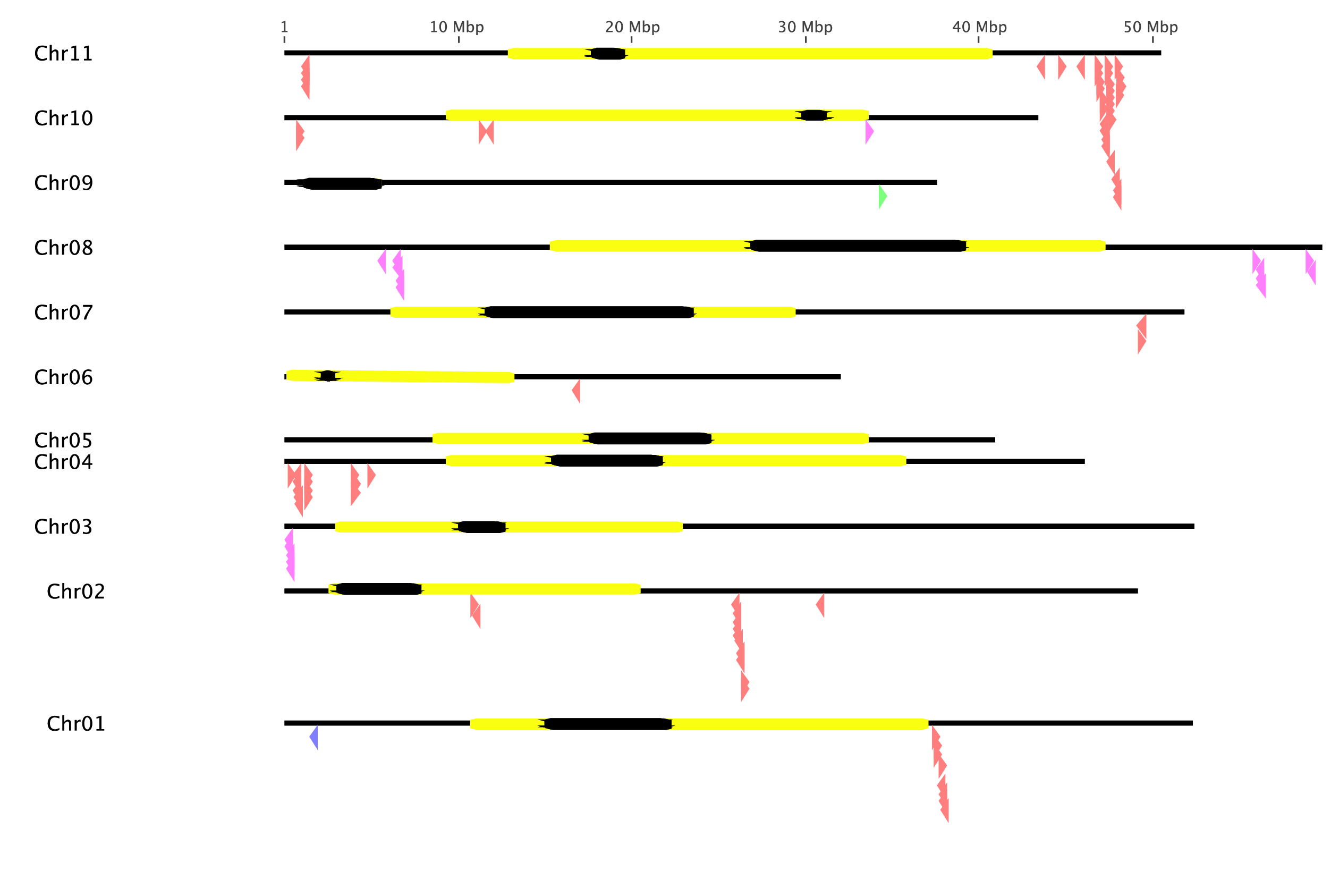

Supplement: Supplementary file 1 [file genes-08-00249-s001.zip › 8_31_17_SupplementaryDocuments V2/Figure S13.tif]

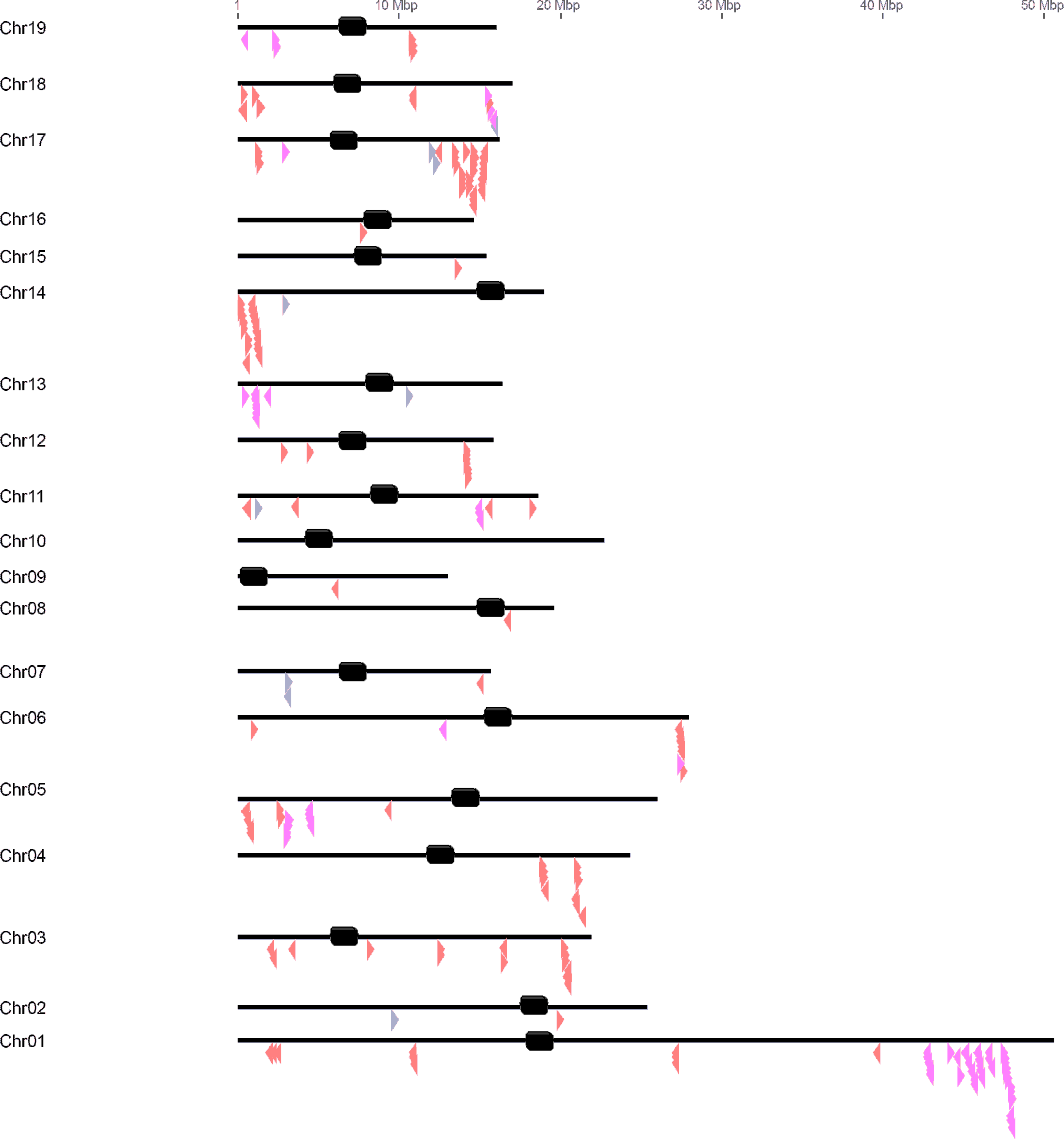

Supplement: Supplementary file 1 [file genes-08-00249-s001.zip › 8_31_17_SupplementaryDocuments V2/Figure S14.tif]

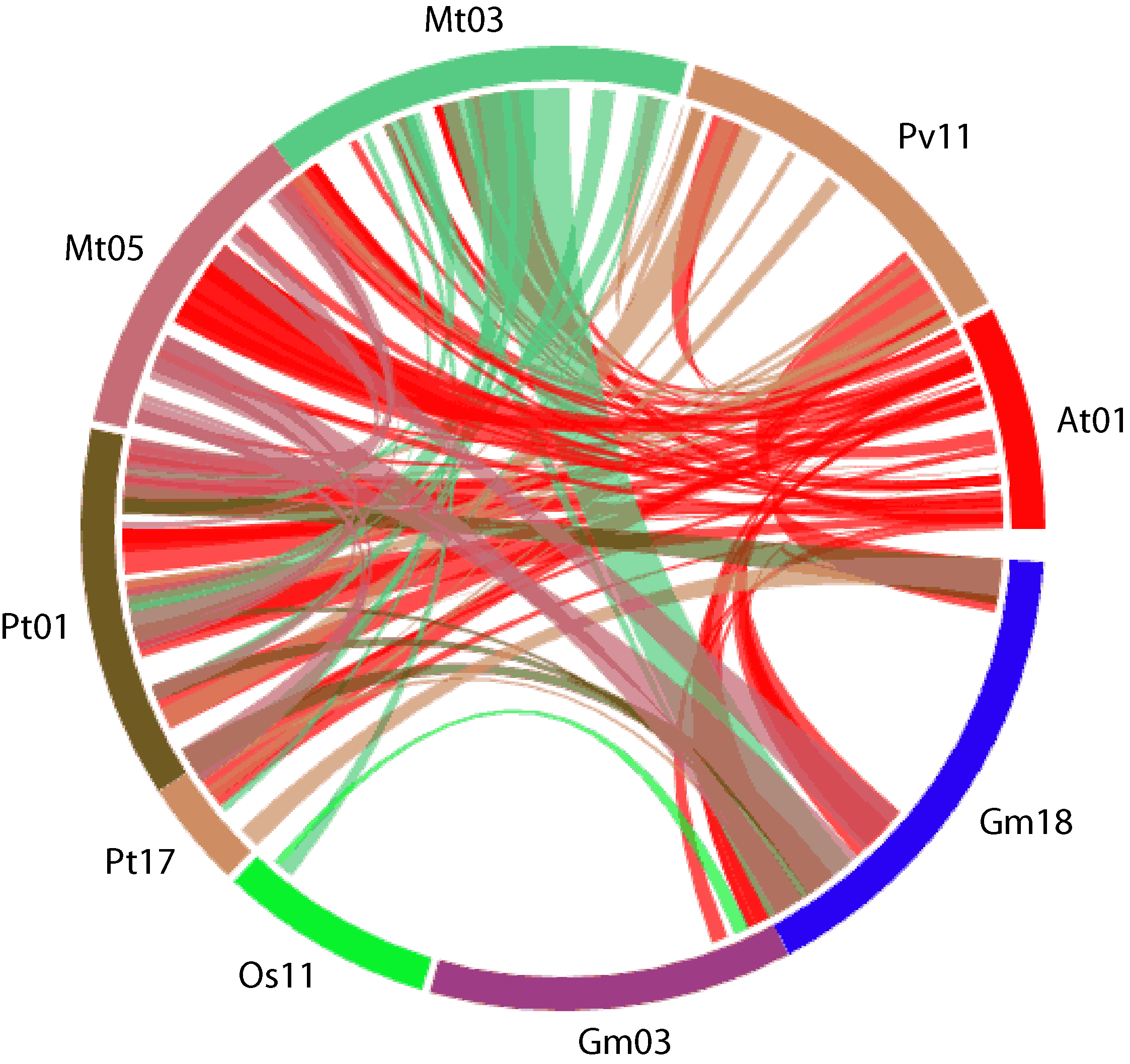

Supplement: Supplementary file 1 [file genes-08-00249-s001.zip › 8_31_17_SupplementaryDocuments V2/Figure S15.tif]

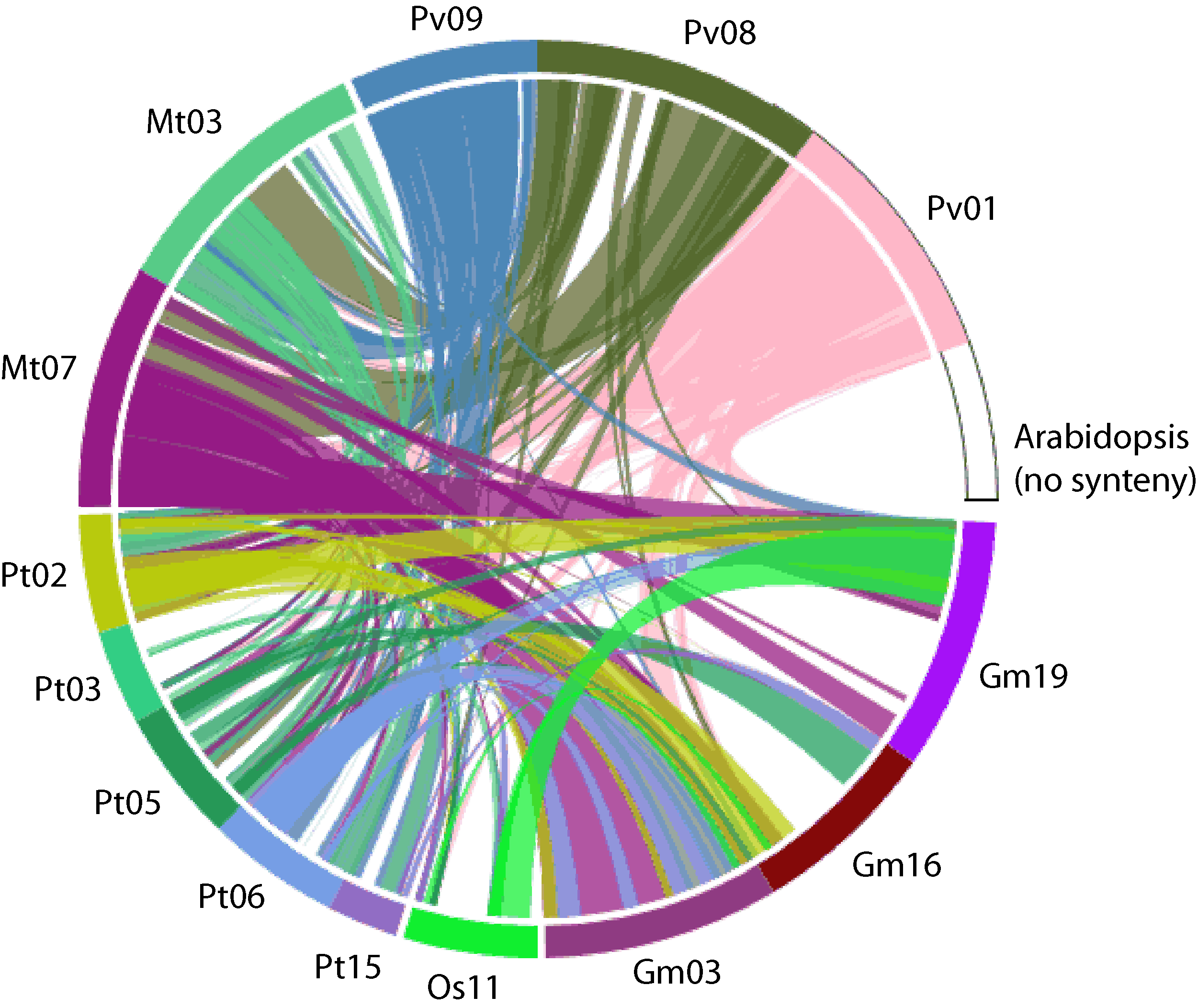

Supplement: Supplementary file 1 [file genes-08-00249-s001.zip › 8_31_17_SupplementaryDocuments V2/Figure S16.tif]

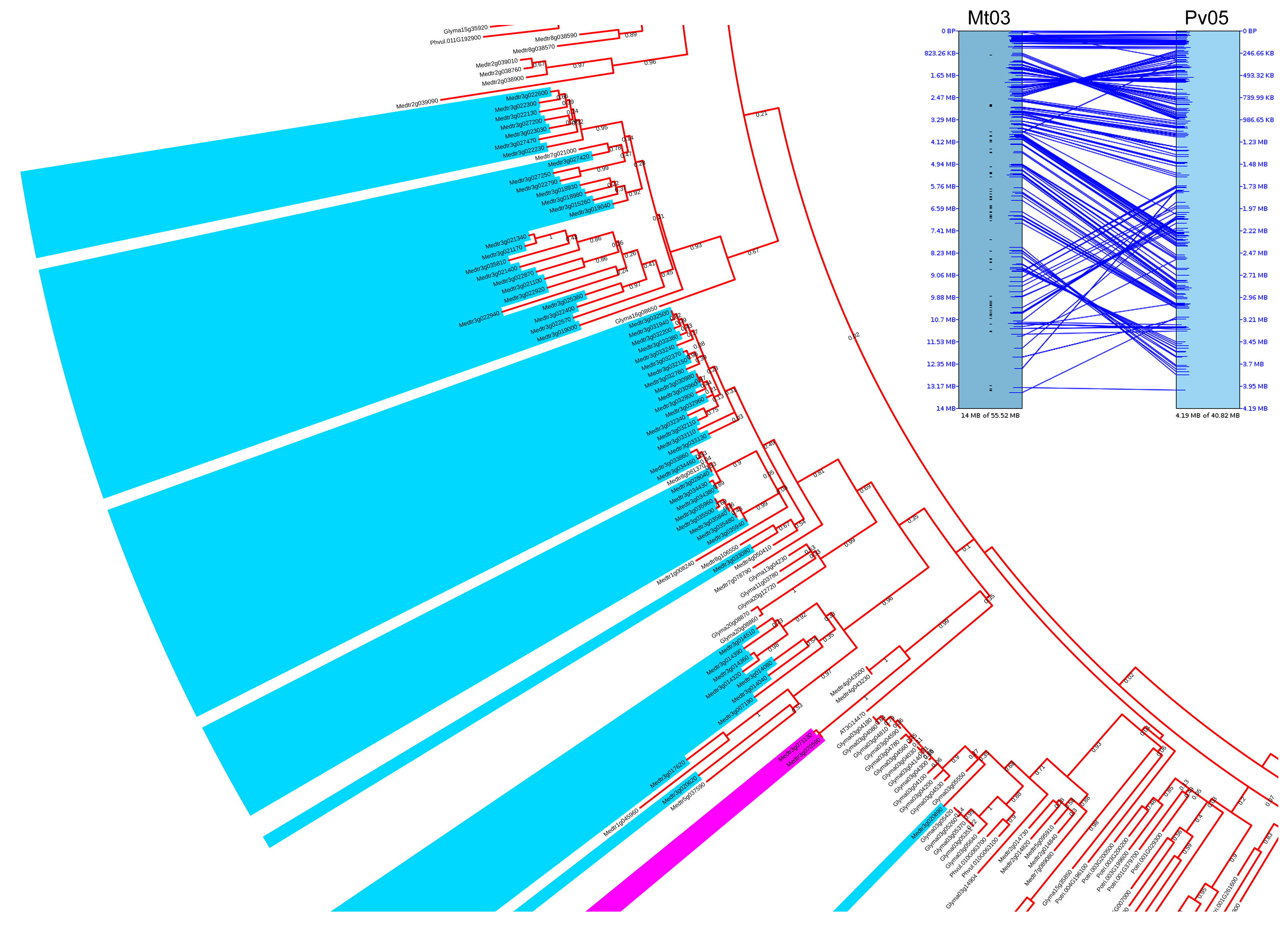

Supplement: Supplementary file 1 [file genes-08-00249-s001.zip › 8_31_17_SupplementaryDocuments V2/Figure S17.tif]

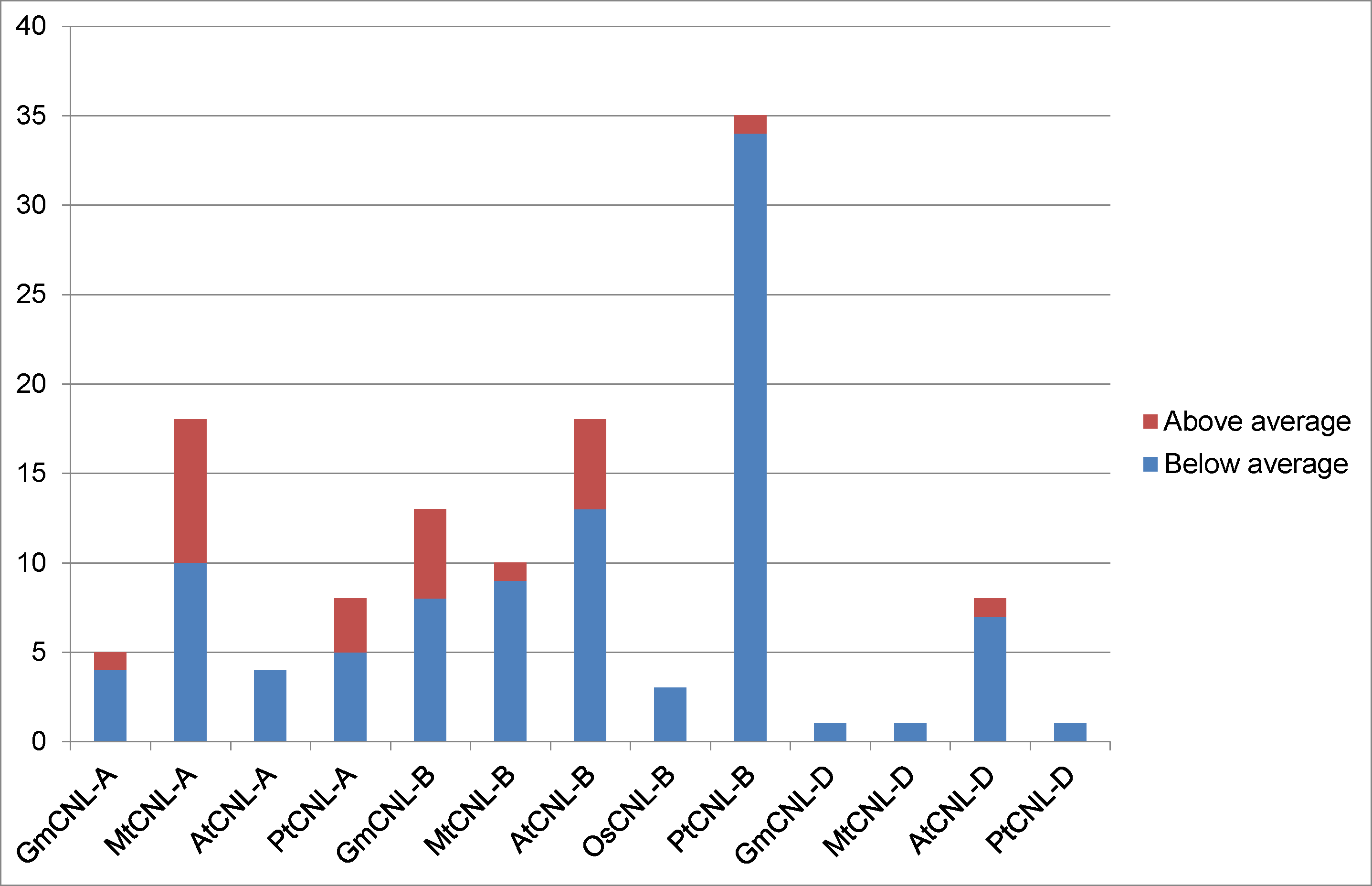

Supplement: Supplementary file 1 [file genes-08-00249-s001.zip › 8_31_17_SupplementaryDocuments V2/Figure S18.tif]

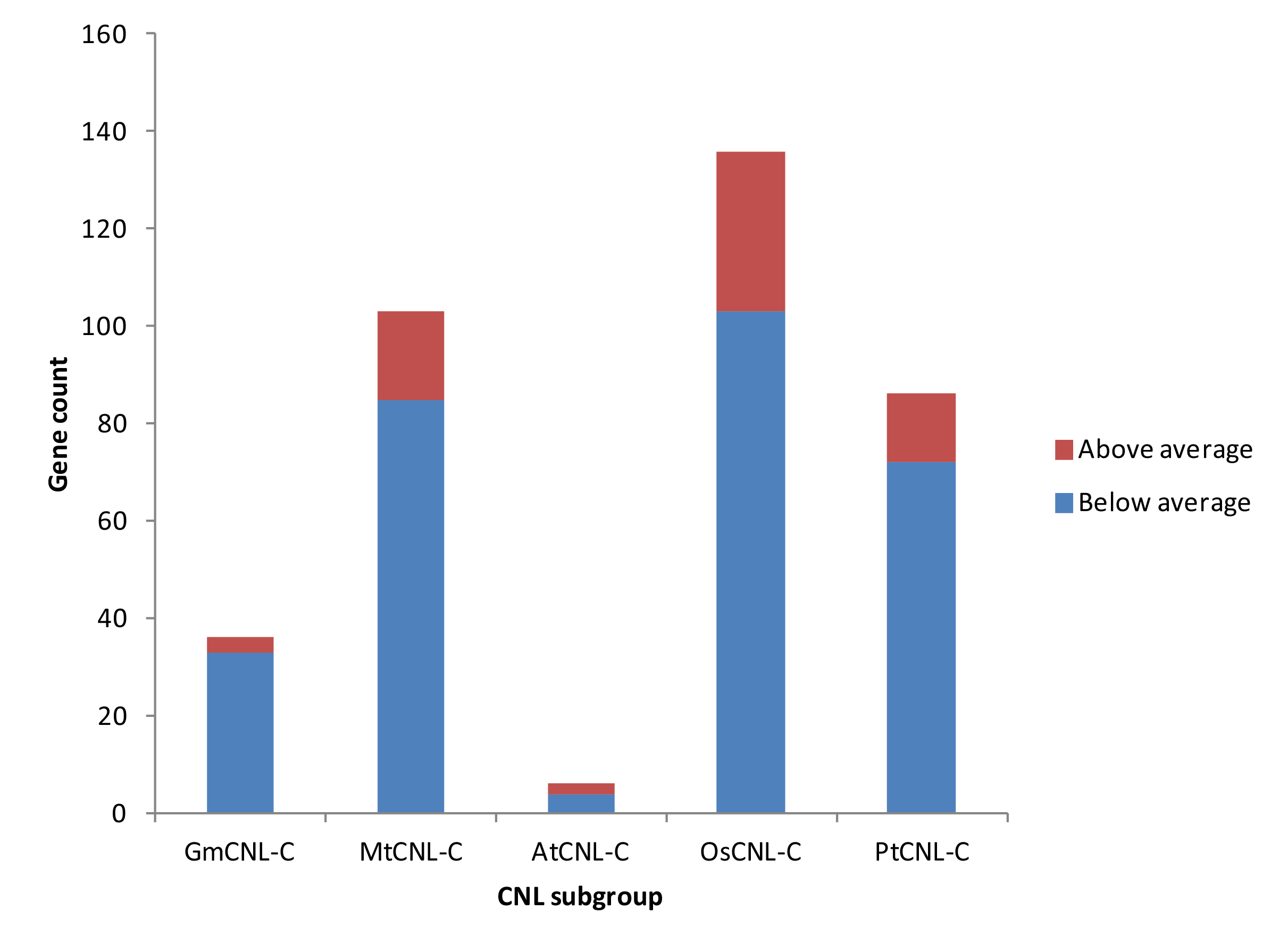

Supplement: Supplementary file 1 [file genes-08-00249-s001.zip › 8_31_17_SupplementaryDocuments V2/Figure S19.tif]

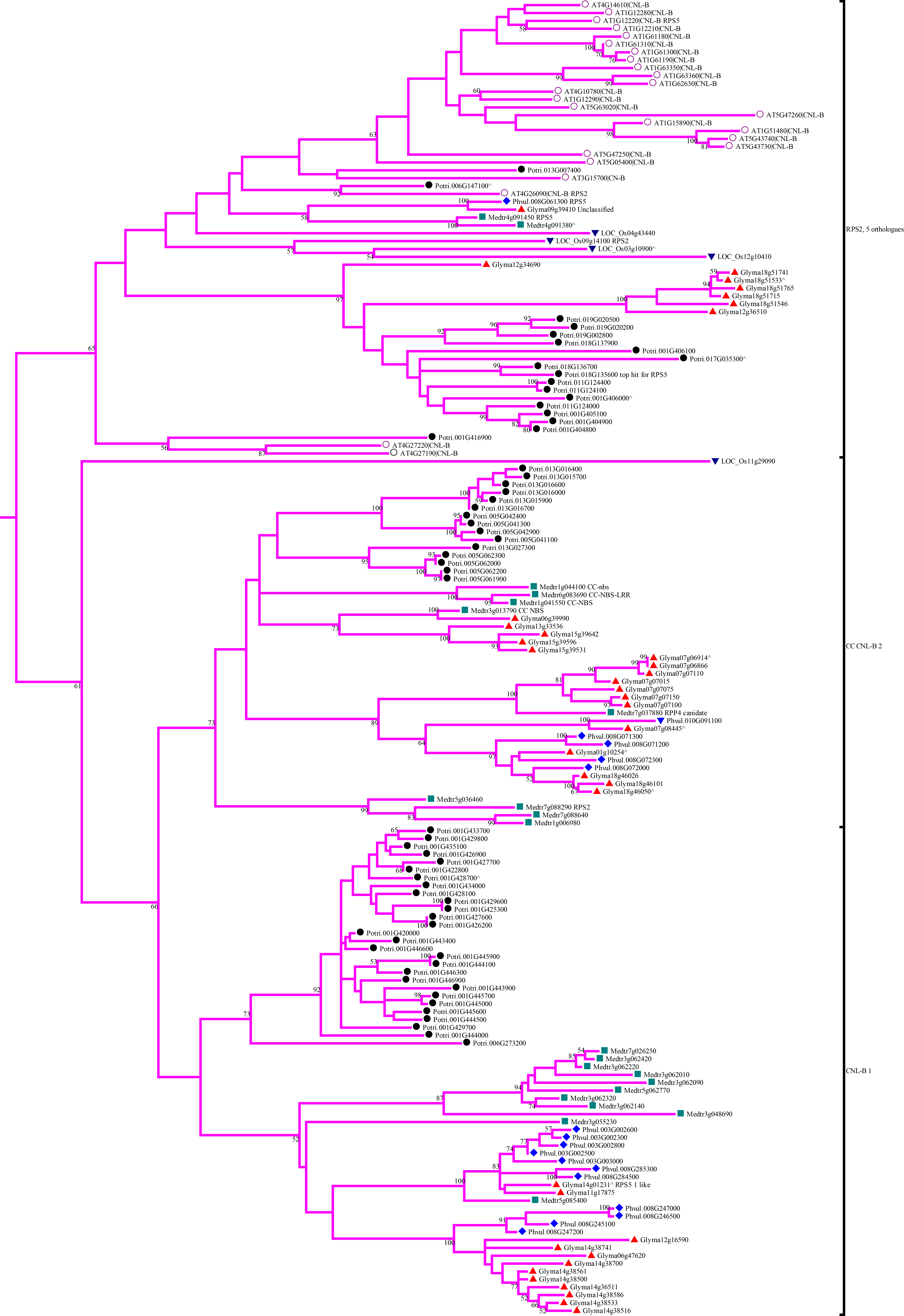

Supplement: Supplementary file 1 [file genes-08-00249-s001.zip › 8_31_17_SupplementaryDocuments V2/Figure S2.tif]

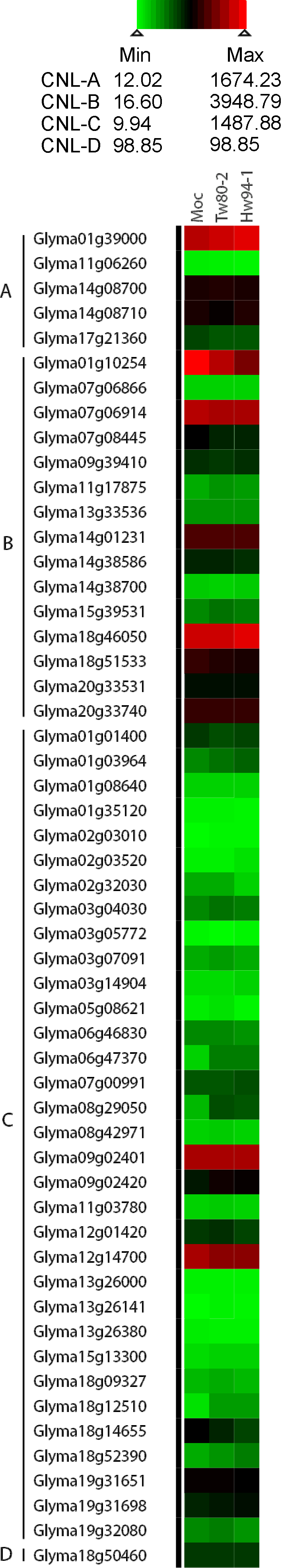

Supplement: Supplementary file 1 [file genes-08-00249-s001.zip › 8_31_17_SupplementaryDocuments V2/Figure S20.tif]

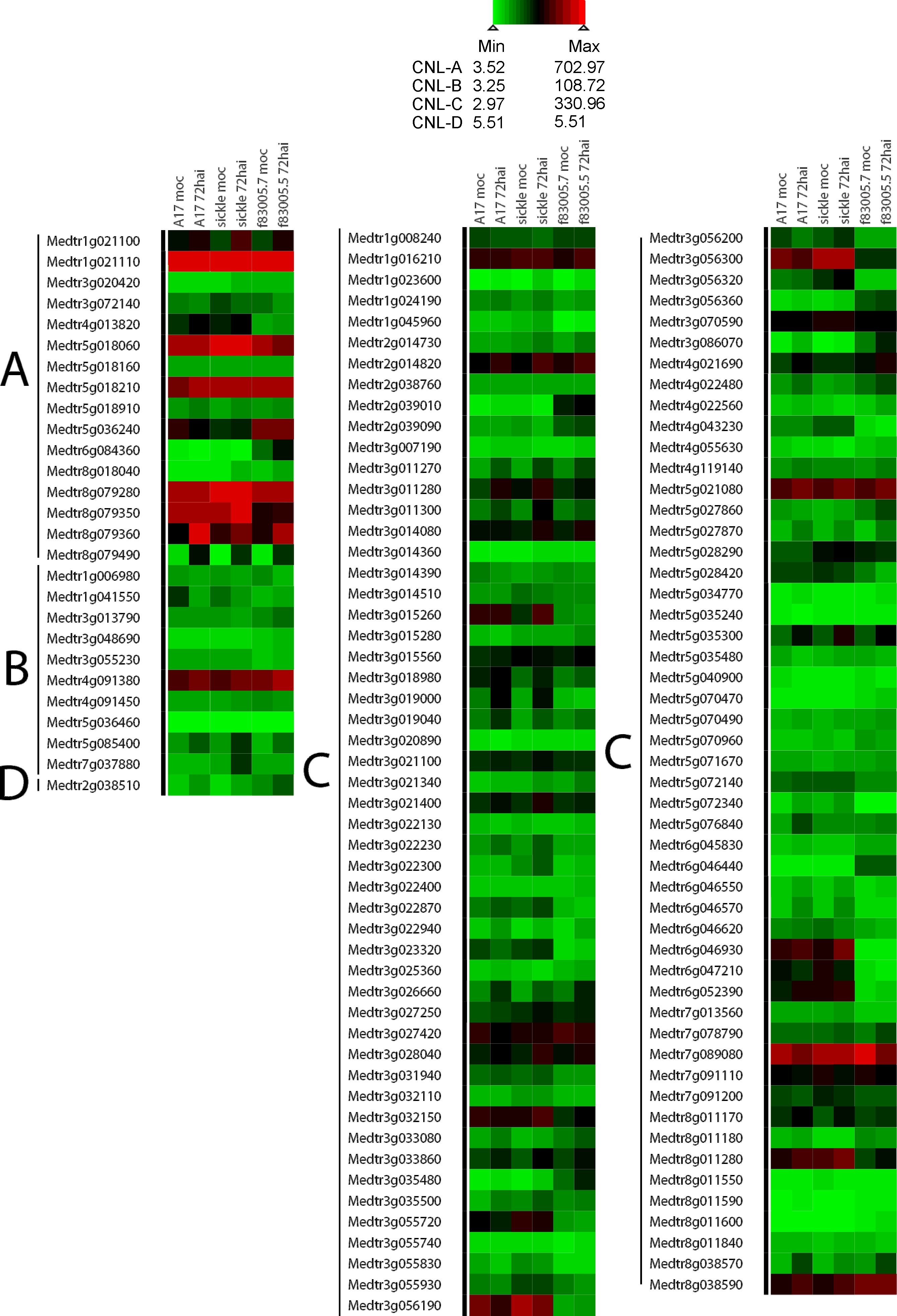

Supplement: Supplementary file 1 [file genes-08-00249-s001.zip › 8_31_17_SupplementaryDocuments V2/Figure S21.tif]

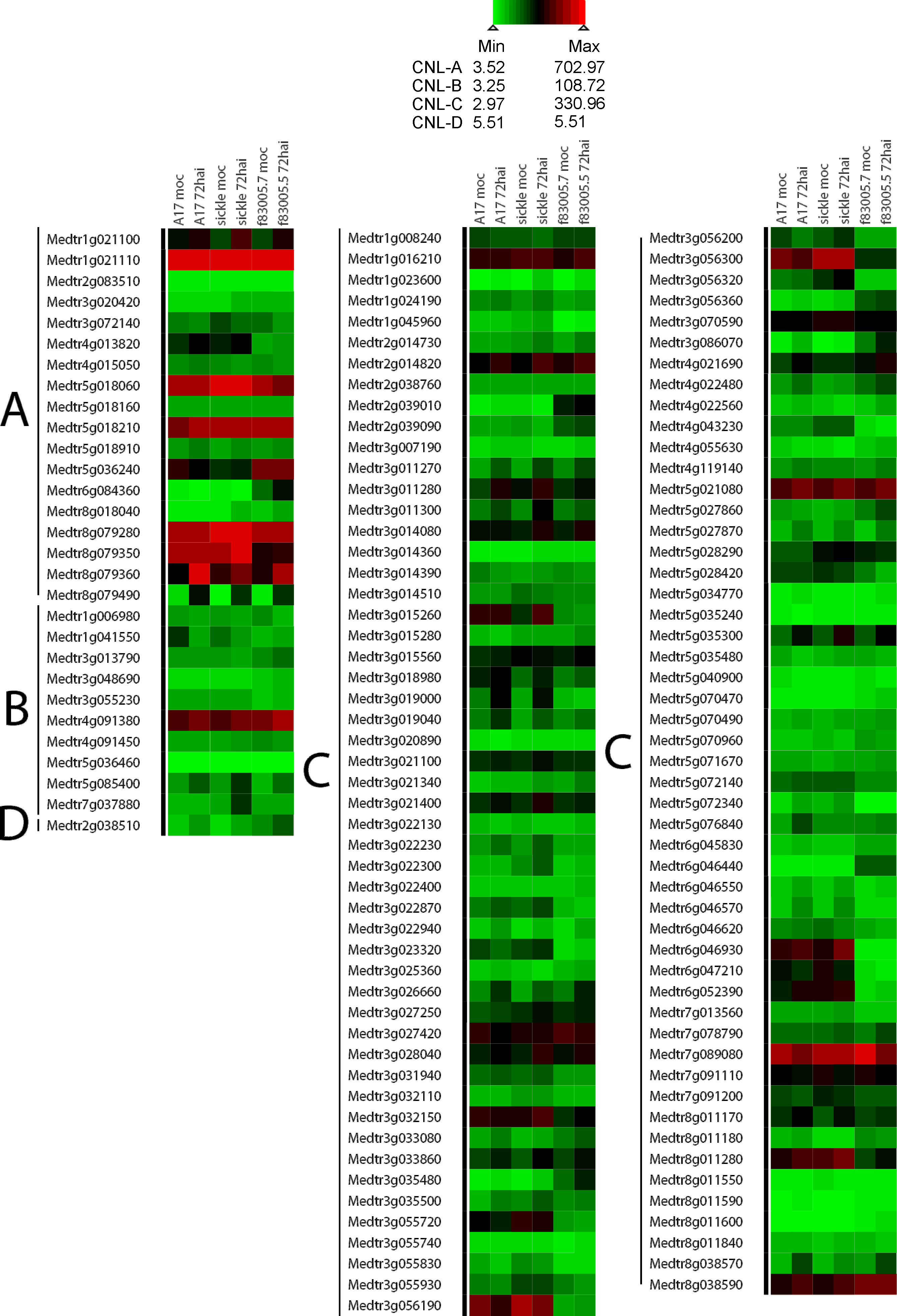

Supplement: Supplementary file 1 [file genes-08-00249-s001.zip › 8_31_17_SupplementaryDocuments V2/Figure S22.tif]

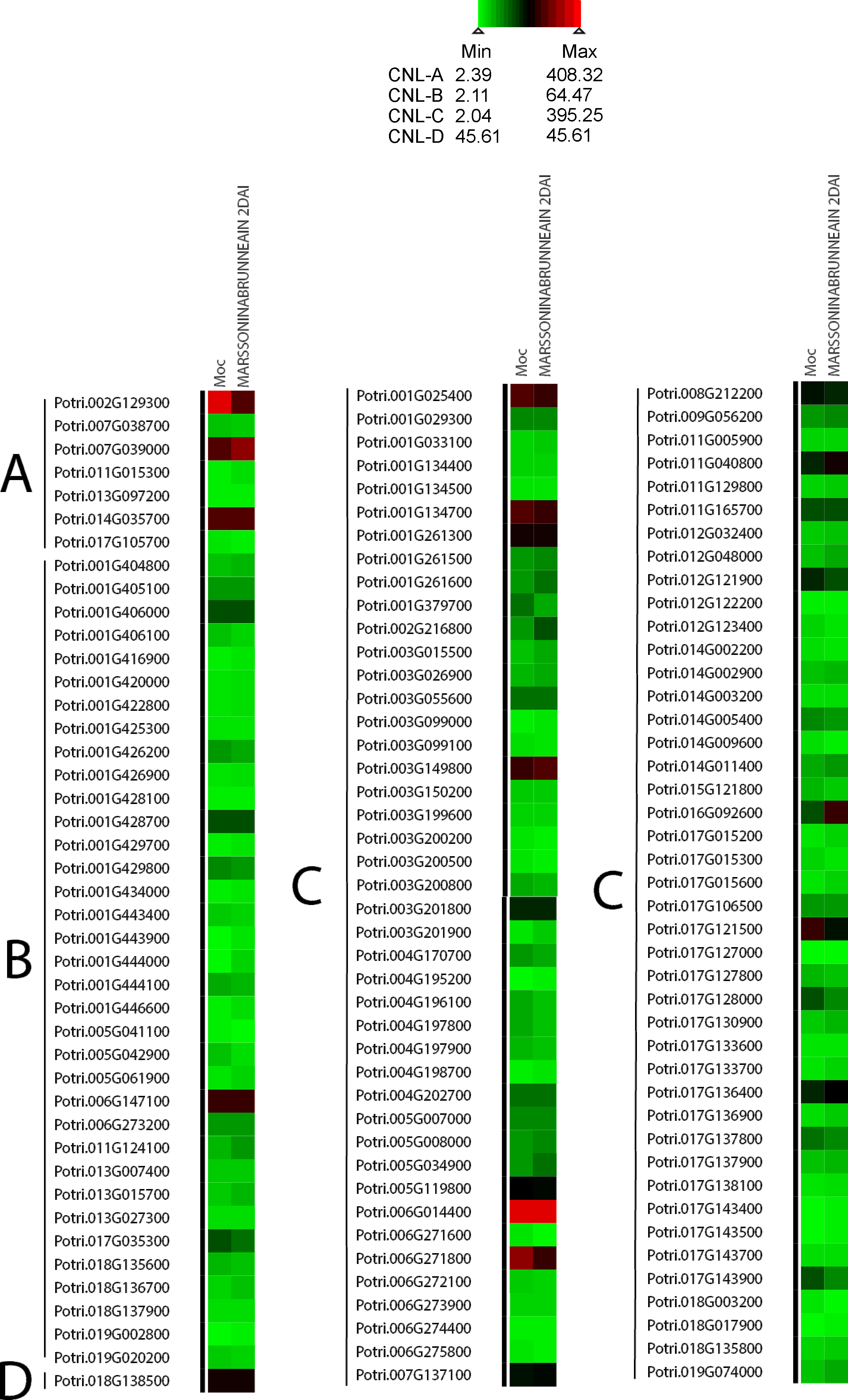

Supplement: Supplementary file 1 [file genes-08-00249-s001.zip › 8_31_17_SupplementaryDocuments V2/Figure S23.tif]

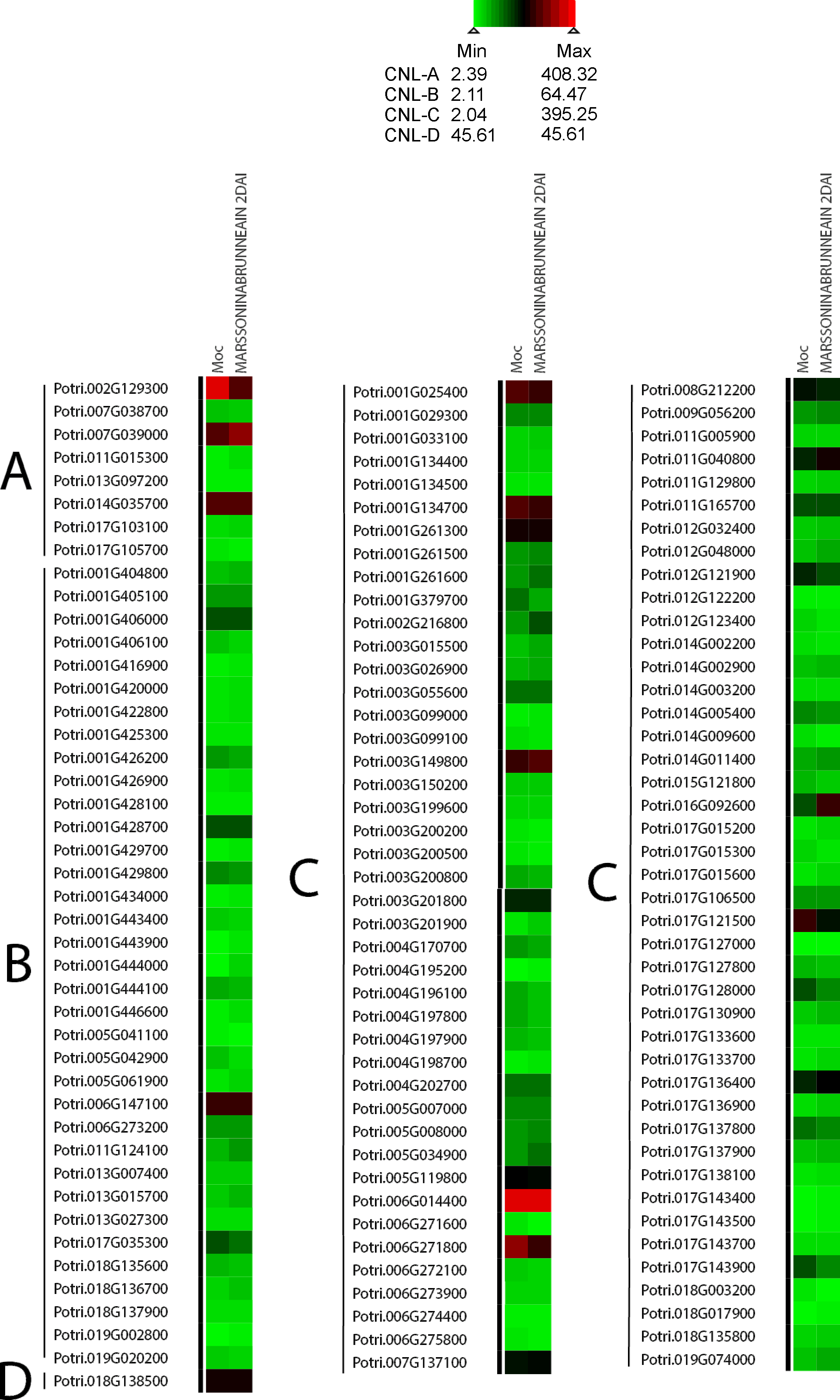

Supplement: Supplementary file 1 [file genes-08-00249-s001.zip › 8_31_17_SupplementaryDocuments V2/Figure S24.tif]

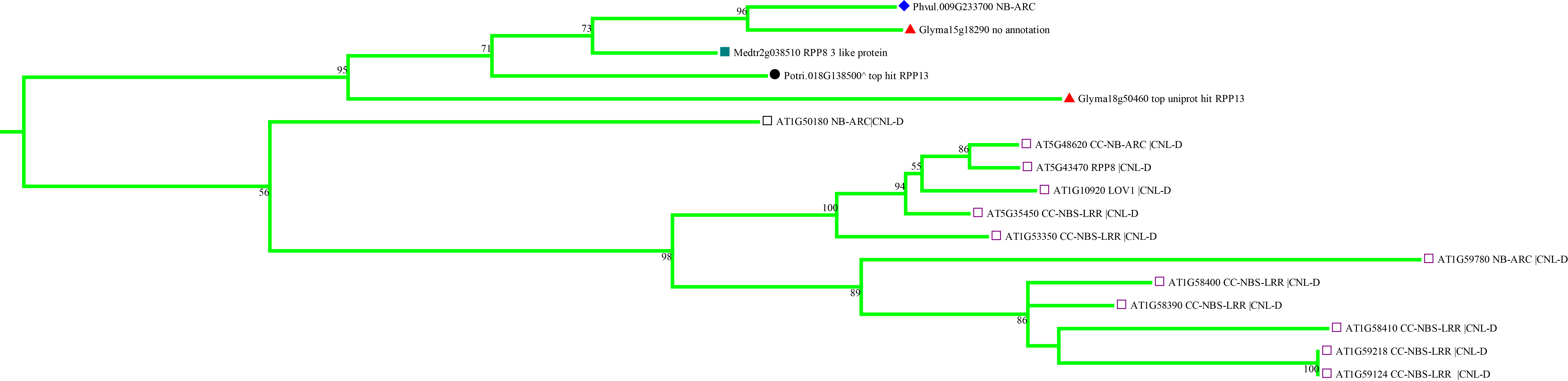

Supplement: Supplementary file 1 [file genes-08-00249-s001.zip › 8_31_17_SupplementaryDocuments V2/Figure S4.tif]

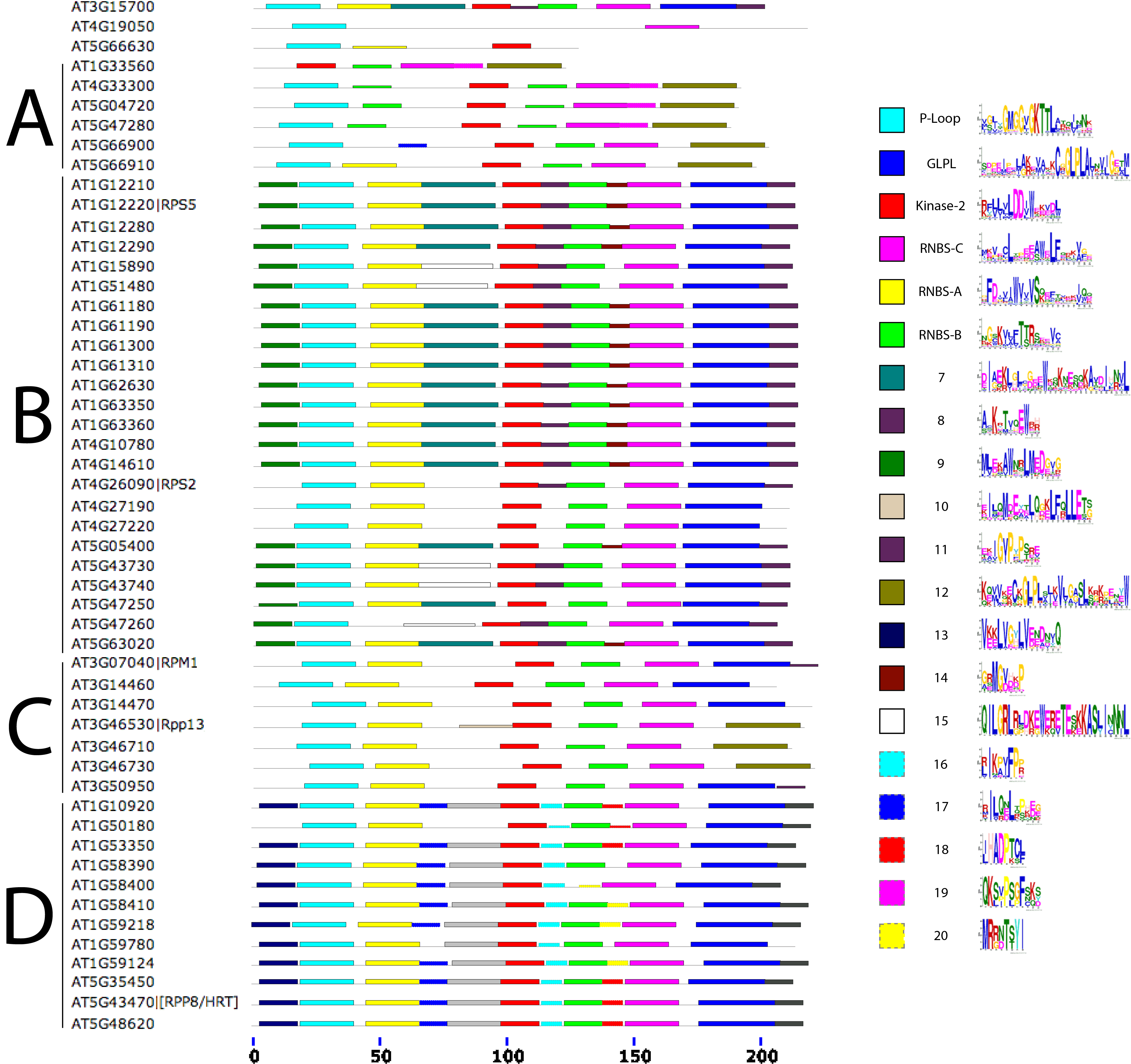

Supplement: Supplementary file 1 [file genes-08-00249-s001.zip › 8_31_17_SupplementaryDocuments V2/Figure S5.tif]

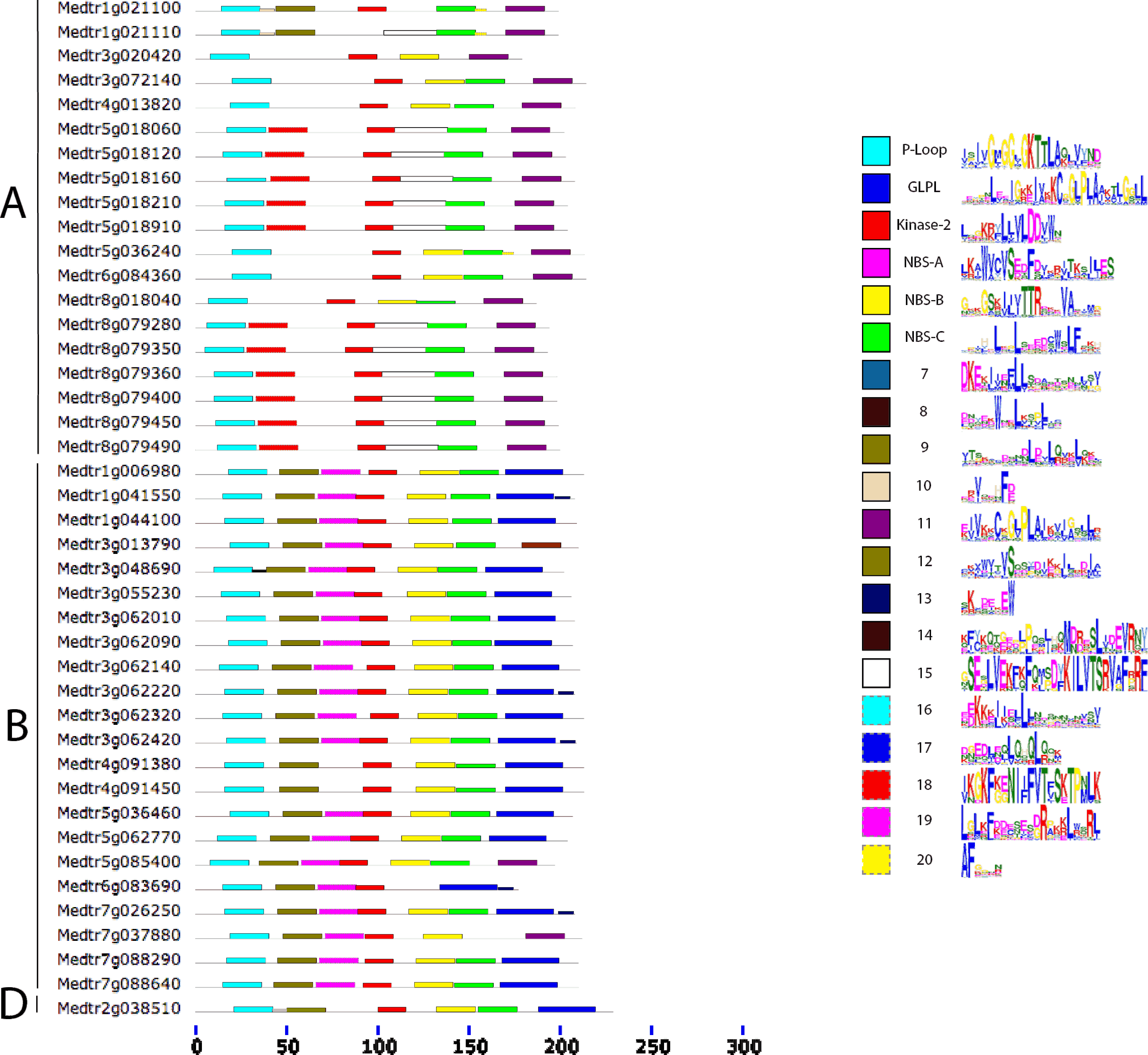

Supplement: Supplementary file 1 [file genes-08-00249-s001.zip › 8_31_17_SupplementaryDocuments V2/Figure S6A.tif]

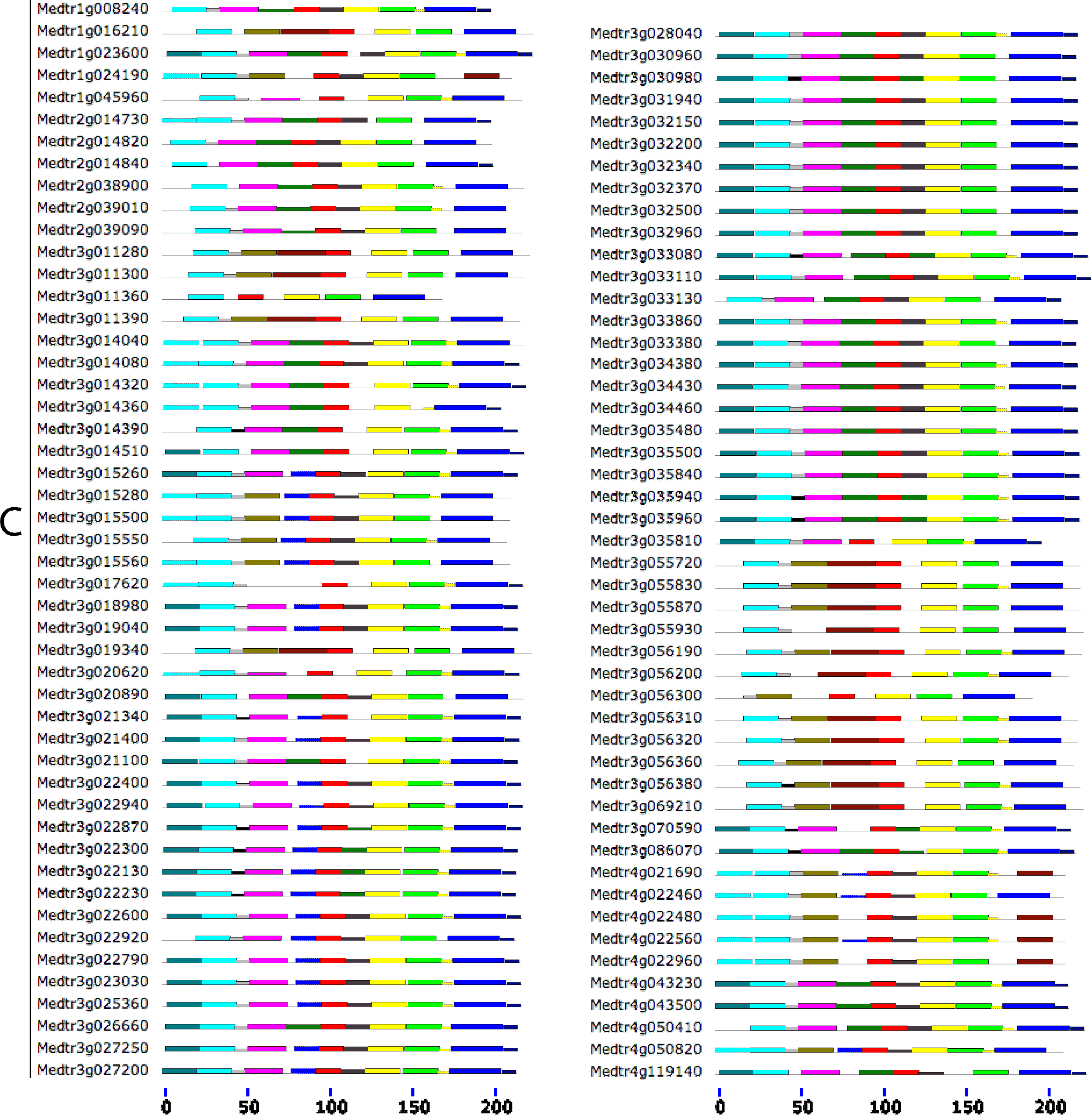

Supplement: Supplementary file 1 [file genes-08-00249-s001.zip › 8_31_17_SupplementaryDocuments V2/Figure S6B.tif]

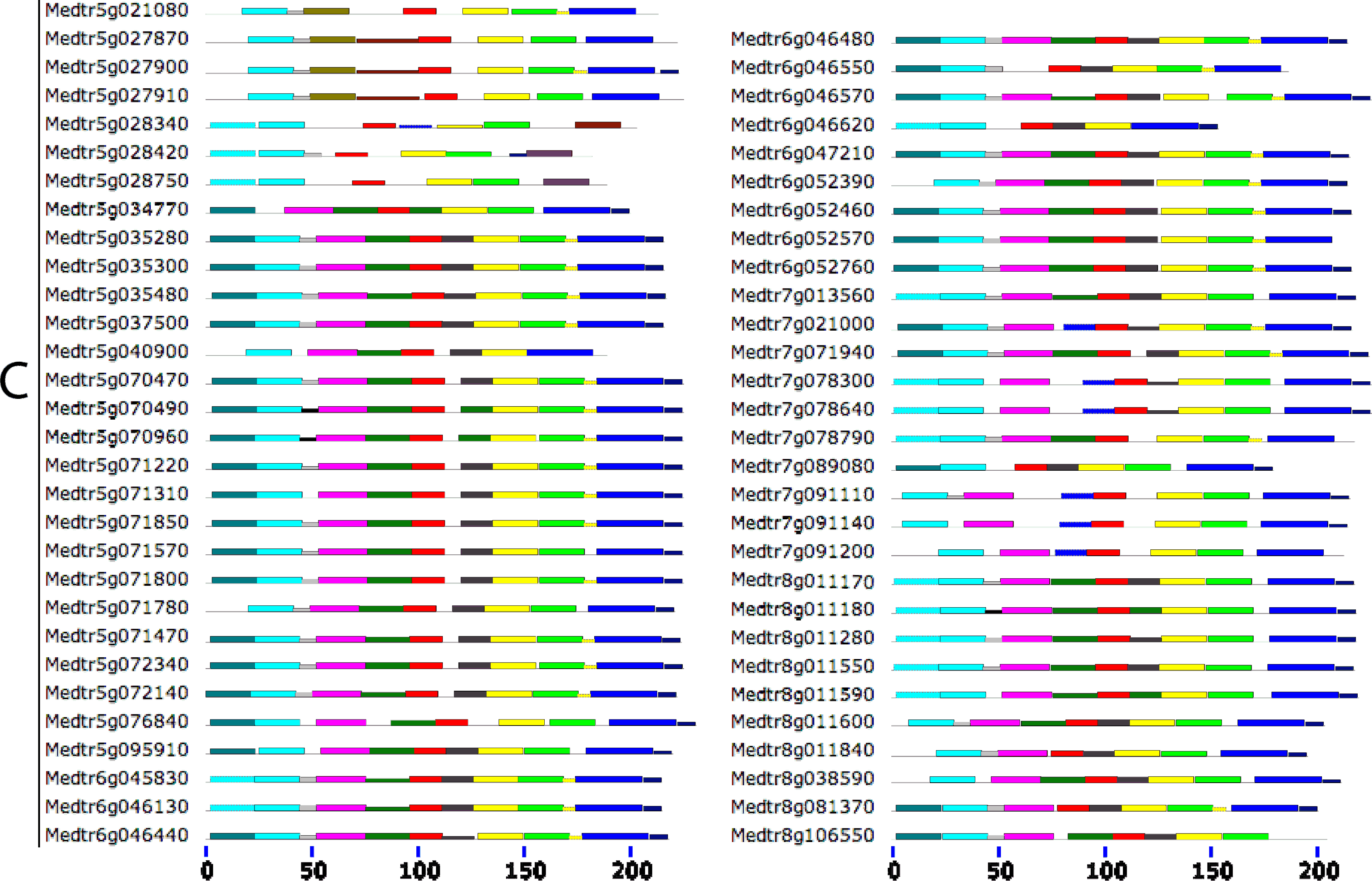

Supplement: Supplementary file 1 [file genes-08-00249-s001.zip › 8_31_17_SupplementaryDocuments V2/Figure S6C.tif]

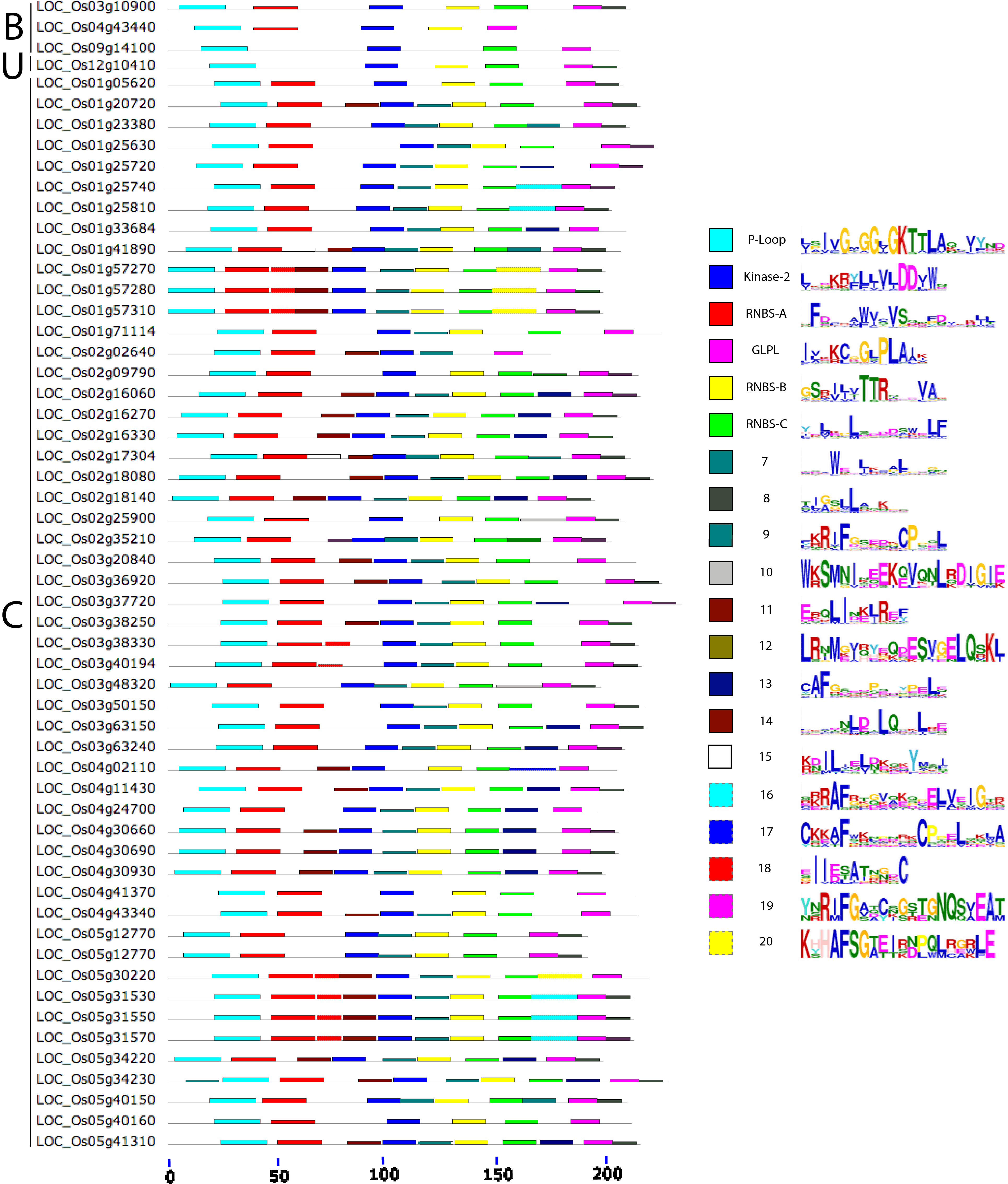

Supplement: Supplementary file 1 [file genes-08-00249-s001.zip › 8_31_17_SupplementaryDocuments V2/Figure S7A.tif]

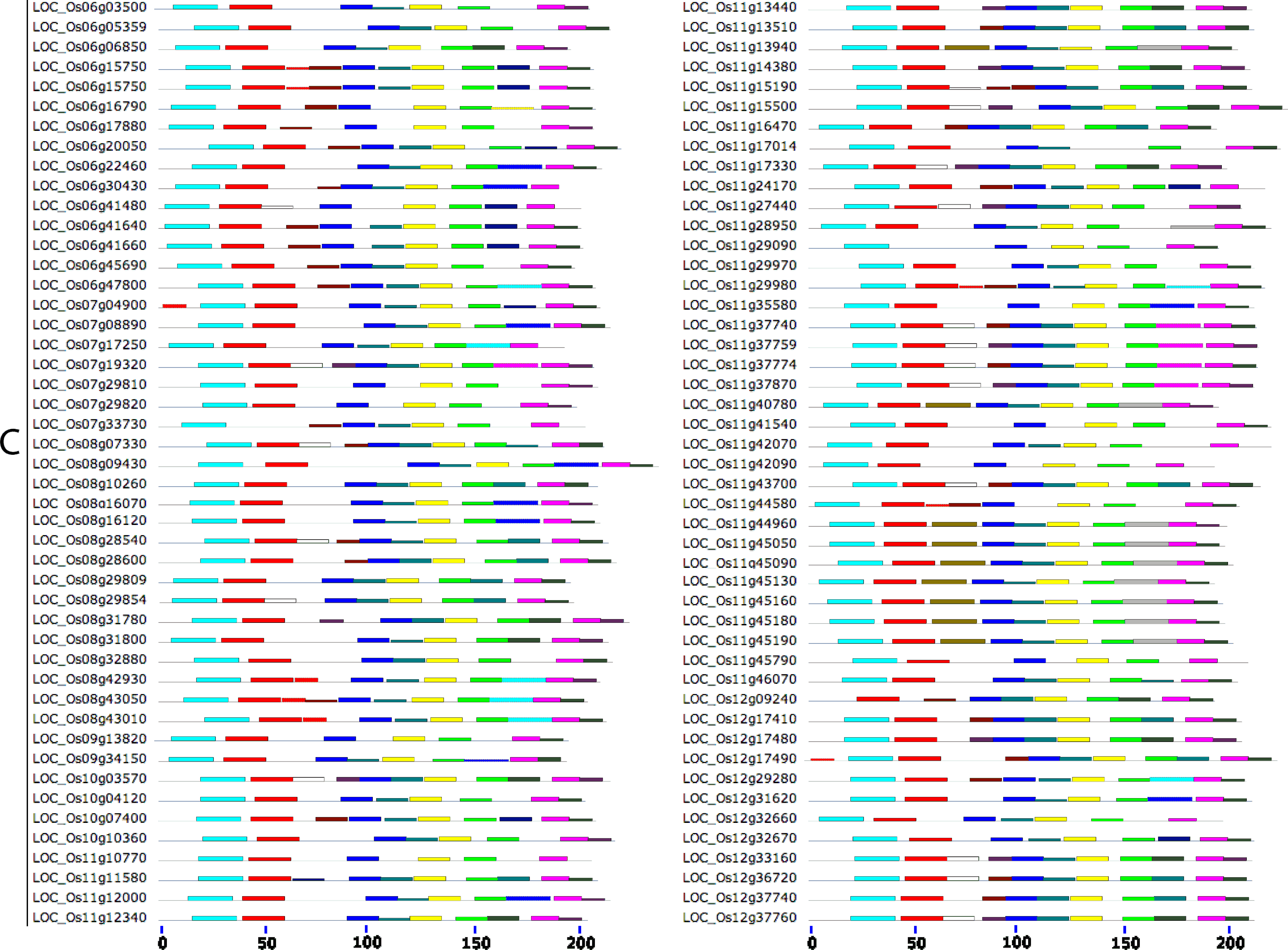

Supplement: Supplementary file 1 [file genes-08-00249-s001.zip › 8_31_17_SupplementaryDocuments V2/Figure S7B.tif]

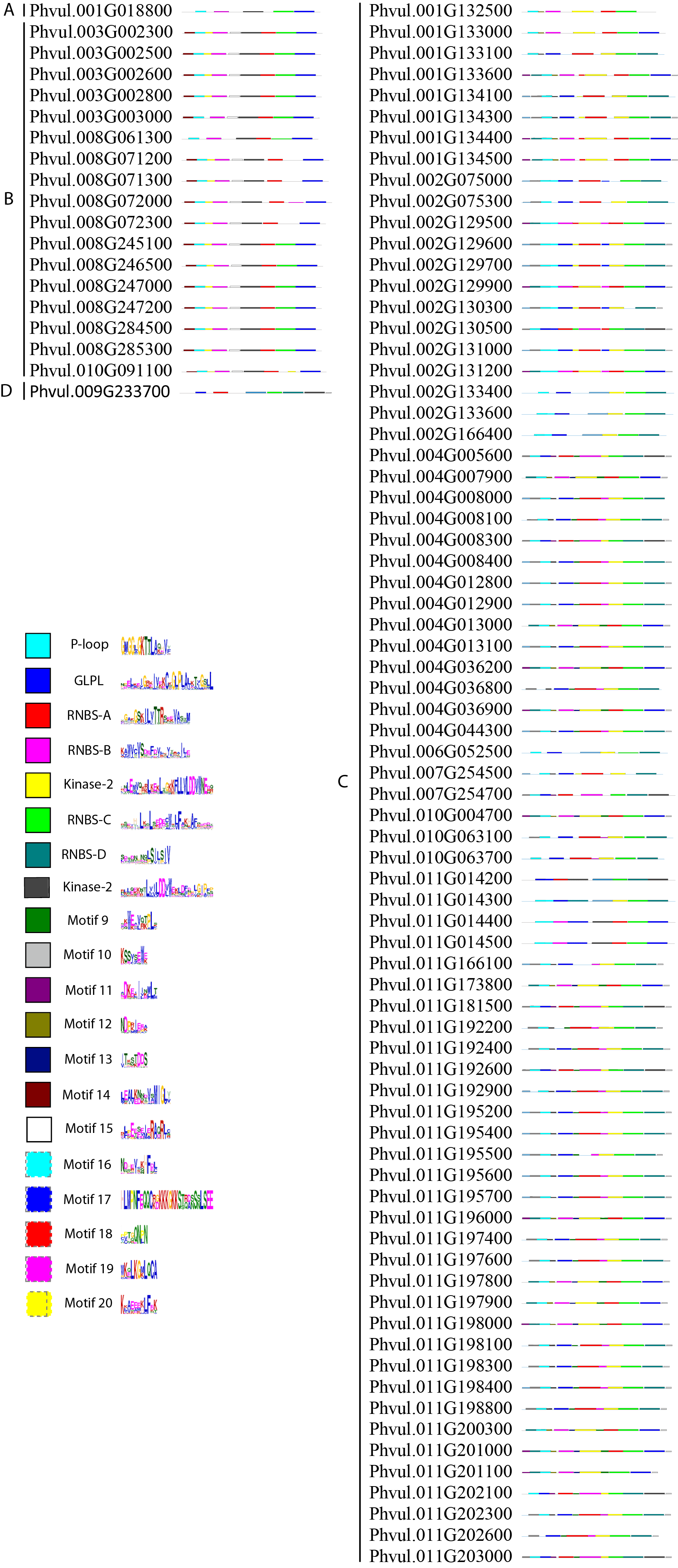

Supplement: Supplementary file 1 [file genes-08-00249-s001.zip › 8_31_17_SupplementaryDocuments V2/Figure S8.tif]

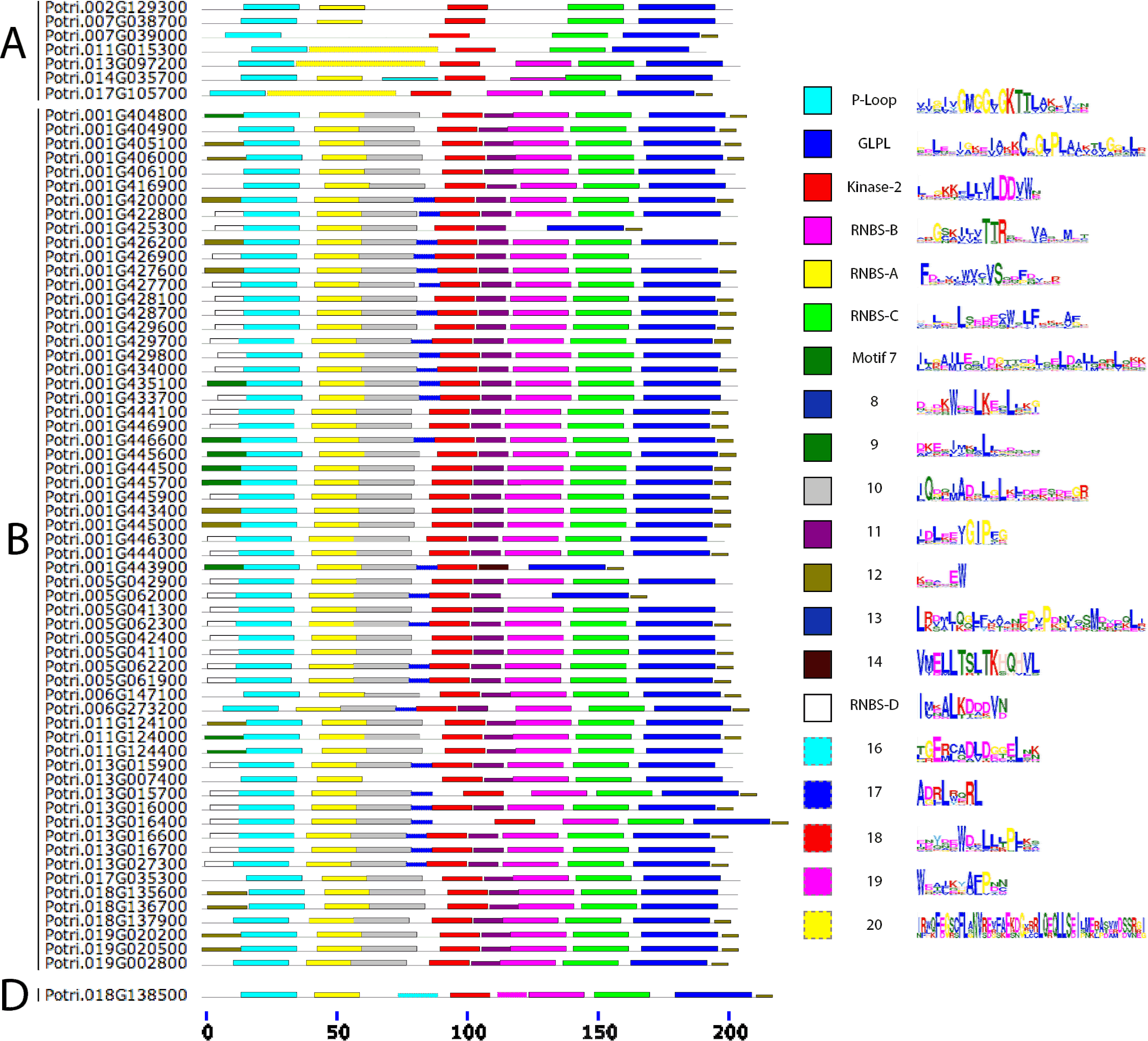

Supplement: Supplementary file 1 [file genes-08-00249-s001.zip › 8_31_17_SupplementaryDocuments V2/Figure S9A.tif]

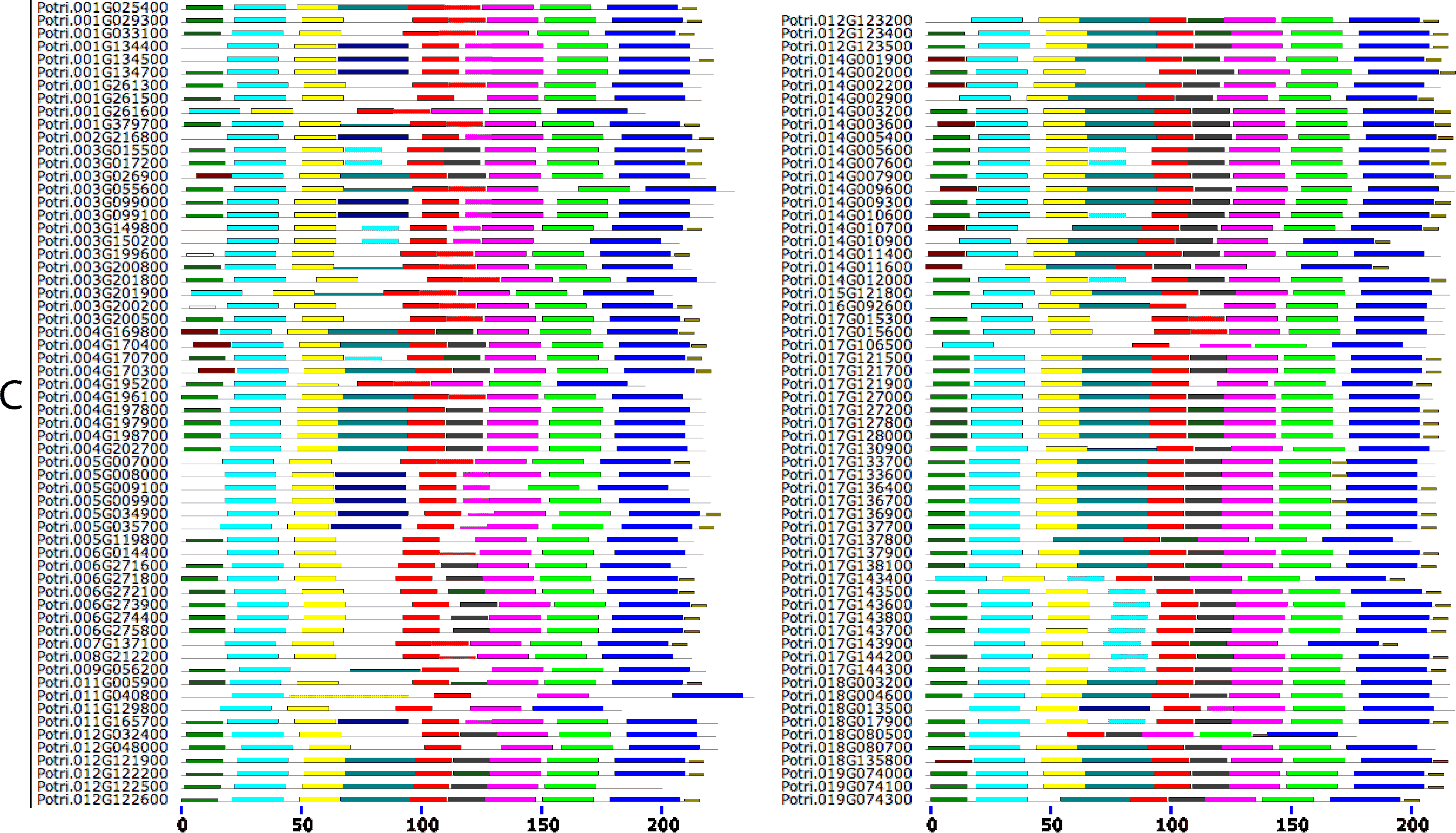

Supplement: Supplementary file 1 [file genes-08-00249-s001.zip › 8_31_17_SupplementaryDocuments V2/Figure S9B.tif]
